# Anatomical Transcriptome of G Protein-Coupled Receptors Leads to the Identification of a Novel Therapeutic Candidate GPR52 for Psychiatric Disorders

**DOI:** 10.1371/journal.pone.0090134

**Published:** 2014-02-28

**Authors:** Hidetoshi Komatsu, Minoru Maruyama, Shuuhei Yao, Tokuyuki Shinohara, Kensuke Sakuma, Sachiko Imaichi, Tomoko Chikatsu, Kanako Kuniyeda, Foo Kok Siu, Lam Sock Peng, Katherine Zhuo, Lay Sock Mun, Tan Min Han, Yoshio Matsumoto, Tadatoshi Hashimoto, Nobuyuki Miyajima, Yasuaki Itoh, Kazuhiro Ogi, Yugo Habata, Masaaki Mori

**Affiliations:** 1 Central Nervous System Drug Discovery Unit, Pharmaceutical Research Division, Takeda Pharmaceutical Company Limited, Fujisawa, Kanagawa, Japan; 2 Cardiovascular and Metabolic Drug Discovery Unit, Pharmaceutical Research Division, Takeda Pharmaceutical Company Limited, Fujisawa, Kanagawa, Japan; 3 Biomolecular Research Laboratories, Pharmaceutical Research Division, Takeda Pharmaceutical Company Limited, Fujisawa, Kanagawa, Japan; 4 Advanced Science Research Laboratories, Pharmaceutical Research Division, Takeda Pharmaceutical Company Limited, Fujisawa, Kanagawa, Japan; 5 Extra Value Generation and General Medicine Drug Discovery Unit, Pharmaceutical Research Division, Takeda Pharmaceutical Company Limited, Fujisawa, Kanagawa, Japan; 6 TSP CNS Phenotyping, Takeda Pharmaceutical Company Limited, Singapore; 7 TSP Transgenic pipeline, Takeda Pharmaceutical Company Limited, Singapore; 8 Research Administration Department, Pharmaceutical Research Division, Takeda Pharmaceutical Company Limited, Fujisawa, Kanagawa, Japan; 9 Pharmaceutical Marketing Division, Takeda Pharmaceutical Company Limited, Fujisawa, Kanagawa, Japan; University of Maryland Schoool of Medicine, United States of America

## Abstract

Many drugs of abuse and most neuropharmacological agents regulate G protein-coupled receptors (GPCRs) in the central nervous system (CNS)_ENREF_1. The striatum, in which dopamine D1 and D2 receptors are enriched, is strongly innervated by the ventral tegmental area (VTA), which is the origin of dopaminergic cell bodies of the mesocorticolimbic dopamine system_ENREF_3 and plays a central role in the development of psychiatric disorders_ENREF_4. Here we report the comprehensive and anatomical transcript profiling of 322 non-odorant GPCRs in mouse tissue by quantitative real-time PCR (qPCR), leading to the identification of neurotherapeutic receptors exclusively expressed in the CNS, especially in the striatum. Among them, GPR6, GPR52, and GPR88, known as orphan GPCRs, were shown to co-localize either with a D2 receptor alone or with both D1 and D2 receptors in neurons of the basal ganglia. Intriguingly, we found that GPR52 was well conserved among vertebrates, is Gs-coupled and responsive to the antipsychotic drug, reserpine. We used three types of transgenic (Tg) mice employing a *Cre-lox* system under the control of the GPR52 promoter, namely, GPR52-LacZ Tg, human GPR52 (hGPR52) Tg, and hGPR52-GFP Tg mice. Detailed histological investigation suggests that GPR52 may modulate dopaminergic and glutamatergic transmission in neuronal circuits responsible for cognitive function and emotion. In support of our prediction, GPR52 knockout and transgenic mice exhibited psychosis-related and antipsychotic-like behaviors, respectively. Therefore, we propose that GPR52 has the potential of being a therapeutic psychiatric receptor. This approach may help identify potential therapeutic targets for CNS diseases.

## Introduction

G protein-coupled receptors (GPCRs) comprise the largest membrane superfamily of signaling molecules and are divided into two categories: odorant/sensory and non-odorant. Odorant/sensory receptors are exposed to the environment and detect external stimuli such as odors, tastes, and pheromones. Non-odorant GPCRs are expressed throughout the whole body, react to a manifold of ligands. They control diverse physiological processes including hemostasis, metabolism, and neurotransmission. Approximately 60% of all drug targets are located on the cell surface membrane. Hence, non-odorant GPCRs comprise one of the most important therapeutic target repertoires and account for 28% of all current drug targets[1].

Intriguingly, many CNS drugs modulate the activity of GPCRs[2]. GPCRs respond to a variety of neurotransmitters by acting through signaling pathways that mediate slow synaptic transmission[3], [4]. Dopamine receptors represent prototypic examples of GPCR mediated neurotransmission[5]. Dopamine is involved in the regulation of locomotion, reward, and affection[6]–[8]. Abnormal dopaminergic neurotransmission is tightly linked with multiple neurological and psychiatric disorders such as Parkinson's disease, Huntington's disease, attention deficit hyperactivity disorder (ADHD), mood disorders, and schizophrenia[3], [9]. GPCRs are well known to play a pivotal role in mitigating several symptoms of schizophrenia. It has been shown in clinical studies that typical and atypical antipsychotics such as haloperidol and olanzapine possess antagonistic activities for the dopamine D2 receptor as well as other multiple GPCRs[10]–[12].

In general, gene expression of GPCRs is found at low levels and often fails to be detected accurately by DNA microarray analysis[13]. Regard et al. reported the tissue profiling of 353 non-odorant GPCR mRNA expressions in 41 tissues from adult mice by quantitative real-time PCR (qPCR) and then attempted to predict physiological functions for individual receptors[14]. Here, we investigate the comprehensive and anatomical expressions of 322 non-odorant GPCR genes in 40 mouse tissues by qPCR and then identify neurotherapeutic receptor candidates exclusively expressed in the CNS, especially in the striatum. The striatum is strongly governed by the VTA and substantia nigra pars compacta (SNc). The VTA is the origin of the dopaminergic mesolimbic pathway that is known be overactive in schizophrenia[15], [16]. Among the receptor candidates expressed in the striatum, GPR52 is a highly conserved, Gs-coupled receptor responsive to the antipsychotics, reserpine. To date, there has been no report of the comprehensive expression profile and biological function of GPR52 since this receptor was identified from human genomic DNA[17]. Through detailed histological investigation and analyses of GPR52 knockout and transgenic mice, we show that GPR52 has the potential of being a therapeutic psychiatric receptor.

## Materials and Methods

### Quantitative expression analysis

Comprehensive GPCR expression analysis of mouse tissues was performed by TaqMan RT-PCR. Total RNA was extracted using ISOGEN (NipponGene) from tissues of adult C57BL/6 or Balb/c mice (pool of 3-17 animals), and cDNAs were synthesized using SuperScript II (Invitrogen). Taqman RT-PCR was carried out using Sequence Detection System Prism 7900HT (Applied Biosystems). Primer and probe of mouse 322 GPCRs were designed and produced by Applied Biosystems (Table S2). Other expression analyses were also performed by TaqMan RT-PCR using primers listed in Table S4. Human and rat tissue cDNA were synthesized from total RNA purchased at Clontech and extracted from tissues of male adult SD-rat (pool of 3–17 animals), respectively. Tissue cDNA of GPR52-Tg mice were also synthesized from total RNA described above. Data are represented as copy number in 1 ng total RNA by duplicate determinations. In some cases, data were normalized by GAPDH or cyclophilin genes.

### Clustering for comprehensive GPCR expression data

To produce the hierarchical clustering of comprehensive GPCR expression data set, MATLAB software (The Mathworks, Natick, MA) was used to perform calculations and to display the results graphically. After converting raw values to log2 ratio (Table S1), Euclidean was applied to calculate gene distances and a hierarchical tree was created based on ward method. Information on GPCR ligands and orphan GPCRs was obtained from Alexander et al[18] or published reports on each GPCR (Table S3).

### Real-time cAMP assay using cyclic nucleotide-gated channels

The cDNA of rat olfactory cyclic nucleotide-gated (CNG) channel (GenBank accession no. X55519) was cloned from rat brain cDNA, and then mutated channels, termed E583M[19], were generated in order to make it more suitable as a cAMP sensor. Mutant channels were subcloned into pcDNA3.1 (+) neo (Invitrogen), and then were used to generate HEK293 clones stably expressing the mutated CNG channels. The expression plasmids carrying GPCRs were transiently transfected into HEK293 cells using lipofectamine standard transfection methods. Cells were seeded into black clear-bottom 96-well plates, incubated overnight, and loaded with Fluo3-acetoxymethylester (Dojin). Addition of drugs and measurement of changes in intracellular calcium were performed using a fluorometric imaging plate reader (FLIPR, Molecular Devices). Library plates with 96-well format contained natural and synthetic bioactive compounds.

### cAMP Production Assay

CHO cells expressing human GPR52, or TGR5 and Mock-CHO cells were established as previously described[20]. Stable clones were incubated with the samples for 20 min in the presence of 0.2 mM IBMX (Sigma). The amount of cAMP was determined with a cAMP-Screen System (Applied Biosystems).

### Receptor internalization assay

CHO cells expressing acGFP-fused hGPR52 were established as previously described[21]. Stable clones were incubated with the samples for 30 min, and the localization of GFP was determined by confocal laser microscopy.

### Mice

The care and use of the animals and the experimental protocols used in this research were approved by the Experimental Animal Care and Use Committee of Takeda Pharmaceutical Company Limited, and the Guide for the Care and Use of Laboratory Animals were maintained throughout the study (Institute of Laboratory Animal Resources, National Academic Press 1996; NIH publication number 85–23, revised 1996). BAC (bacterial artificial chromosome) transgenic GPR52 promoter-*Cre* lines were produced by integrating mouse GPR52 promoter-*Cre* clones into C57BL/6J mice (mGPR52-*Cre* Tg). 129/SvEv mice with ROSA26-neo-LacZ reporter, ubiquitin (Ubc) promoter-neo-hGPR52, and Ubc promoter-neo-hGPR52-AcGFP constructs were produced by inserting constructs into ROSA26 site. These Tg mice with 129/SvEv genetic background were backcrossed once or three times with C57BL/6J strains, and then finally mated with the mGPR52-*Cre* Tg (116F line) in which the Cre expressions reflected endogenous GPR52 distribution throughout tissues and were coexpressed with D2 receptors but D1 receptors in the striatum (data not shown). GPR52 KO mice were generated on 129SvEv background and 129SvEv controls were used. Knock out of the gene was confirmed by PCR with primers listed in Table S5. All mice were given *ad libitum* food and water and maintained on a 12:12-h light/dark cycle with lights on at 7:00 am. All animal experiments were carried out in accordance with the rules and regulations of the Takeda Animal Care and Use Committee.

### Measurement of locomotor activity

Measurement of methamphetamine-induced hyperactivity was used an automated activity monitoring system (MDC-LT, BrainScience idea). Activity was evaluated under controlled conditions of room light. Room temperature and humidity were maintained at 23±3°C and 55±15%, respectively. On the test day, mice were acclimated in the test cage individually for 60 min. Mice were s.c. injected with vehicle or 2 mg/kg methamphetamine and immediately returned to the test cage. Locomotor activities were calculated with a computer and expressed as means and standard errors. Each 5-min activity was plotted as a function of time. The total activities before and after methamphetamine injection for 60 and 90 min were calculated.

### Open Field Test

The apparatus for open field consisted of a square arena measuring 60 cm×60 cm enclosed by high, clear plastic walls. The arena was divided into 3 zones; central zone (20 cm×20 cm), intermediate zone (40 cm×40 cm) and peripheral zone (60×60 cm). The mice were habituated to the procedure room with background noise for 60 minutes prior to the test. Mice were placed in the central zone at the start of the test and allowed to freely explore the apparatus for 5 minutes. The apparatus was cleaned with 70% ethanol between subjects. A camera mounted on the ceiling was used for automated tracking and scoring of time spent and distance travelled in each zone by SMART© version 2.5 (Panlab s.l.).

### Prepulse Inhibition (PPI) Test

Sensorimotor gating was tested by measuring the startle response and PPI of the startle response with a SR-LAB startle response system (SD Instruments). Mice were habituated for 1 hour to white noise (65dB) and dosed by intraperitoneal injection with saline, amphetamine (10 mg/kg) and MK-801 (0.2 mg/kg), with 1 week in between treatments in a Latin-squared design. 30 minutes after dosing, mice were placed in a transparent acrylic cylinder connected to a startle detector. They were then subjected to a random series of prepulses of 68, 72 and 74 dB paired with an acoustic startle stimulus of 115 dB. The percent PPI was calculated as follows: ([(startle response to pulse alone) – (startle response to prepulse + pulse)]/startle response to pulse alone) x 100.

### X-Gal histochemistry

Fresh frozen sections (16 µm) were fixed for 30 min with ice-cold 0.5% glutaraldehyde and 2 mM MgCl_2_ in phosphate buffered saline (PBS); washed three times in buffer A (2 mM MgCl_2_, 5 mM EGTA, 0.01% sodium deoxycholate and 0.02% NP40 in PBS) for 5 min each at room temperature; and then stained in the dark in 5 mM potassium-ferricyanide, 5 mM potassium-ferrocyanide and 1 mg/ml X-gal in buffer A at 37°C.

### *In situ* hybridization (ISH)

For single ISH, coronal fresh-frozen sections (16 µm) were prepared. These sections were fixed with 4% paraformaldehyde in 0.1 M phosphate buffer (pH 7.4). The cDNA fragments of genes were cloned into the pCR-BluntII-TOPO vector (Invitrogen). The digoxygenin (DIG)- and fluorescein-labeled riboprobes were produced using these plasmids as templates for *in vitro* transcription. Fixed-sections were acetylated, and incubated in a hybridization buffer containing DIG or fluorescein-labeled riboprobes at 60°C. The sections were washed, treated with alkaline phosphatase-conjugated anti-DIG antibody (Roche) and then visualized by 4-nitroblue tetrazolium chloride (NBT) and 5-bromo-4-chloro-3-indolyl phosphate (BCIP) as blue signals. In order to carrying out double-ISH study, the sections treated as described above were incubated with anti-FITC antibody HRP conjugate (Perkin Elmer), and treated with CSA II (DAKO) to detect fluorescein-labeled riboprobe as FITC signals (Green) or 3, 3′-diaminobenzidine (DAB) signals (Brown). After processing, the sections were mounted, and examined by light or confocal laser microscopy. NBT/BCIP signals were changed to red under imaging software as necessary.

### Immunohistochemistry and GFP observation

Mice were transcardially perfused with 4% paraformaldehyde in 0.1 M phosphate buffer (pH 7.4), and the brains were postfixed for 24 h at 4°C. Free floating brain sections (40 µm) of GPR52-GFP Tg mice were mounted on glass slides, and GFP-signals examined by confocal laser microscopy. For double labeling of GFP-signal and immunohistochemistry, free floating sections (40 µm) were incubated with rabbit anti-D2 receptor antibody (Chemicon), and then visualized by 2nd antibody labeled with Alexa 488 (Invitrogen).

### Statistical analysis

All experiments were performed with a minimum of two independent biological replicates. Statistical analysis was performed as follows. First, the data were tested by Bartlett's test for homogeneity of variance. When the variances were homogeneous, Williams' test was performed. When the variances were heterogeneous, the Shirley-Williams test was performed. Bartlett's test was conducted at the significance level of 0.05, and the Williams and Shirley-Williams tests were conducted at the one-tailed significance level of 0.025. Student's t-test was also performed for statistical analysis as indicated in Figure legends.

## Results and Discussion

### Anatomical expression profiles of non-odorant GPCRs are revealed by qPCR

**Figure 1:**
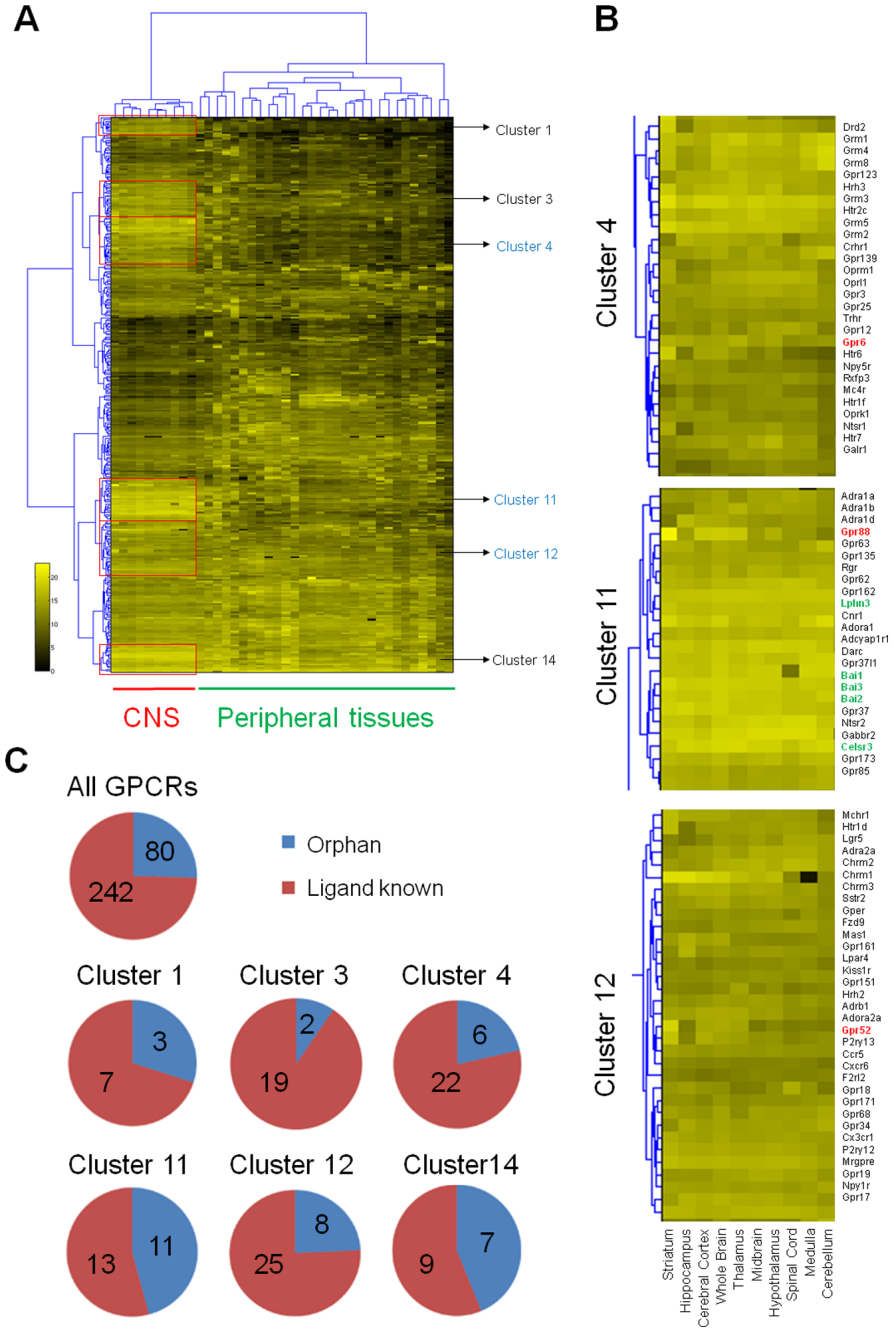
Anatomically comprehensive profiling of mouse GPCR mRNA expression reveals the CNS specific clusters. **A,** Six CNS specific clusters were highlighted by red square. **B,** Cluster 4, 11, and 12 indicated by red numbers in (**A**) were enlarged. Red and Green indicates orphan GPCRs of our interest and adhesion GPCRs, respectively. **C,** Pie charts of the numbers of orphan and ligand-known GPCRs in all and six CNS specific clusters.

We first quantified absolute copy numbers of the transcripts of 322 non-odorant receptors throughout 40 tissues of C57BL/6 mice by quantitative real-time PCR (qPCR) (Figure 1A and Tables S1 and S2). This result is generally consistent with previous reports[14], suggesting that our extensive expression profiling of GPCRs is reliable. By using Euclidean-ward methods for clustering, we found 6 clusters of GPCRs, namely, Clusters 1, 3, 4, 11, 12, and 14, that displayed rich and relatively specific mRNA expression in the CNS, and covered approximately 40% of total non-odorant GPCRs (Figures 1A-B and S1). Approximately one fourth of all examined GPCRs were orphan receptors (Figure 1C and Table S3), however, almost half of Cluster 11 and 14 were orphan receptors (Figure 1C). Cluster 11 exhibited the highest transcript level in the CNS compared to the other clusters and included 5 out of 20 examined adhesion GPCRs. These receptors are known for the ability to facilitate cell and matrix interactions, suggesting that this cluster could be involved in developmental processes in the brain[22] (Figure 1B).

**Figure 2:**
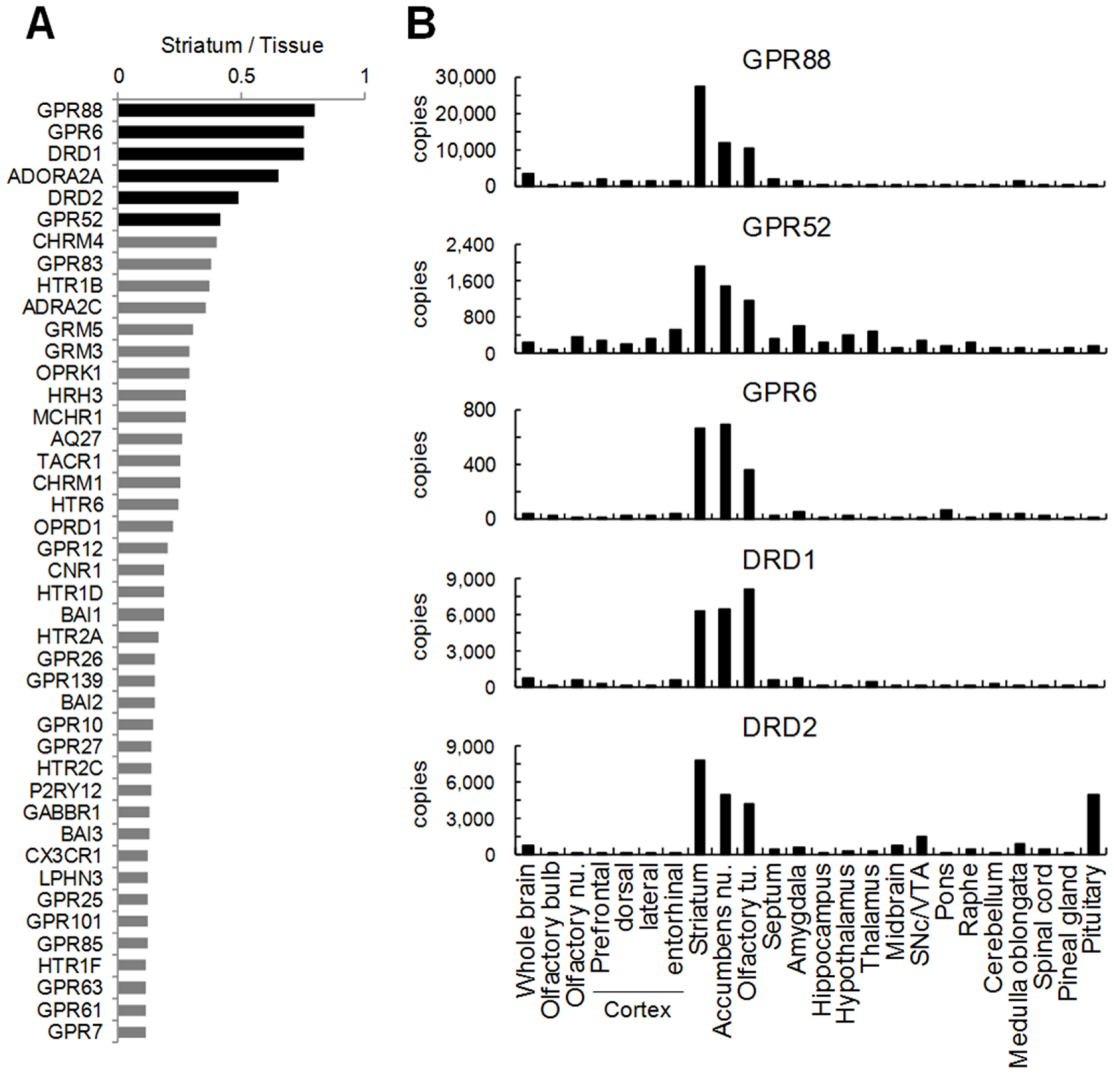
Gene expression of GPCRs enriched in striatum. **A,** Descending order of ratio of mouse striatal expression per total of all the examined tissue expressions for each GPCR was presented using data in Table S1. **B,** Rat brain expressions of the 3 orphan GPCRs and D1 and D2 receptors were quantified by qPCR.

The striatum is an ideal area of investigation for therapeutic targets of psychiatric disorders as it is the largest component of the basal ganglia in which D1 and D2 receptors are most abundant[10], [23], [24]. It also plays a pivotal role in dopaminergic transmission from the VTA and the substantia nigra pars compacta (SNc)[15]. We searched for GPCRs abundantly expressed in mouse striatum using our transcriptional data (Figure 2A), and found that GPR88, GPR6, and GPR52, as well as dopamine D1 and D2 receptors and adenosine A2a receptor (ADORA2A) were the most highly enriched (Figure 2A). GPR6 is an orphan GPCR, although a putative ligand has been identified[25], no ligands have been discovered for GPR52 and GPR88. We confirmed that these 3 receptors were expressed at high levels in rat striatum, showing almost identical expression profiles to those of D1 and D2 receptors (Figure 2B). GPR88, which exhibits remarkably high expression in the brain, belongs to Cluster 11 while GPR6 and GPR52 belong to Cluster 4 (which includes a D2 receptor) and Cluster 12 (which includes an A2a receptor), respectively (Figure 1B).

### GPR52 is expressed in the D2 expressing-MSNs and is a Gs-coupled receptor activated by antipsychotic drug reserpine

**Figure 3:**
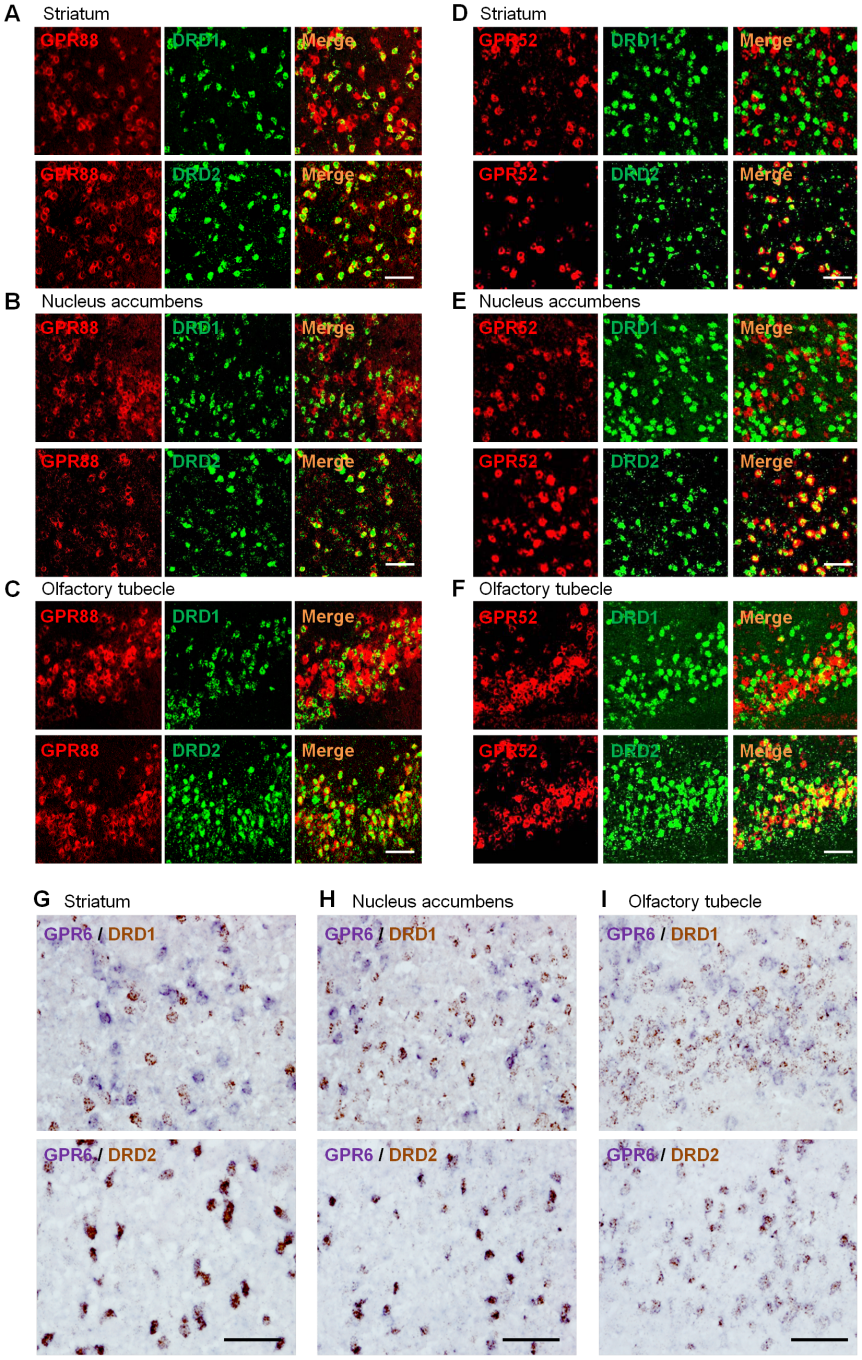
Expression of GPR88, GPR52, and GPR6 in DRD1 or DRD2- expressing neurons of rat basal ganglion by Double-ISH analysis. A–C, GPR88 (red) and DRD1/DRD2 (green) in striatum (A), nucleus accumbens (shell) (B), and olfactory tubecle (C). D–F, GPR52 (red) and DRD1/DRD2 (green) in striatum (D), nucleus accumbens (shell) (E), and olfactory tubecle (F). G–I, GPR6 (blue) and DRD1/DRD2 (brown) in striatum (G), nucleus accumbens (shell) (H), and olfactory tubecle (I). Bar: 100 µm.

Striatal GABAergic medium spiny neurons (MSNs) are believed to regulate processes that are tightly linked with many neuropsychiatric diseases[16]. MSNs constitute the striatonigral (direct) and striatopallidal (indirect) pathways, expressing D1 and D2 receptors, respectively[16], [23]. *In situ* hybridization (ISH) analysis of rat striatum, nucleus accumbens, and olfactory tubercle showed that GPR88 was colocalized with both D1 and D2 receptors (Figure 3A–C)_ENREF_14, whereas GPR52 and GPR6 were colocalized with the D2, but not with the D1 receptor (Figure 3D–I). These data for GPR88 and GPR6 were consistent with previous reports[26]–[28]. Taken together, our data suggest that these 3 orphan GPCRs may play a pivotal role in dopaminergic transmission and be attractive therapeutic targets for psychiatric disorders.

**Figure 4:**
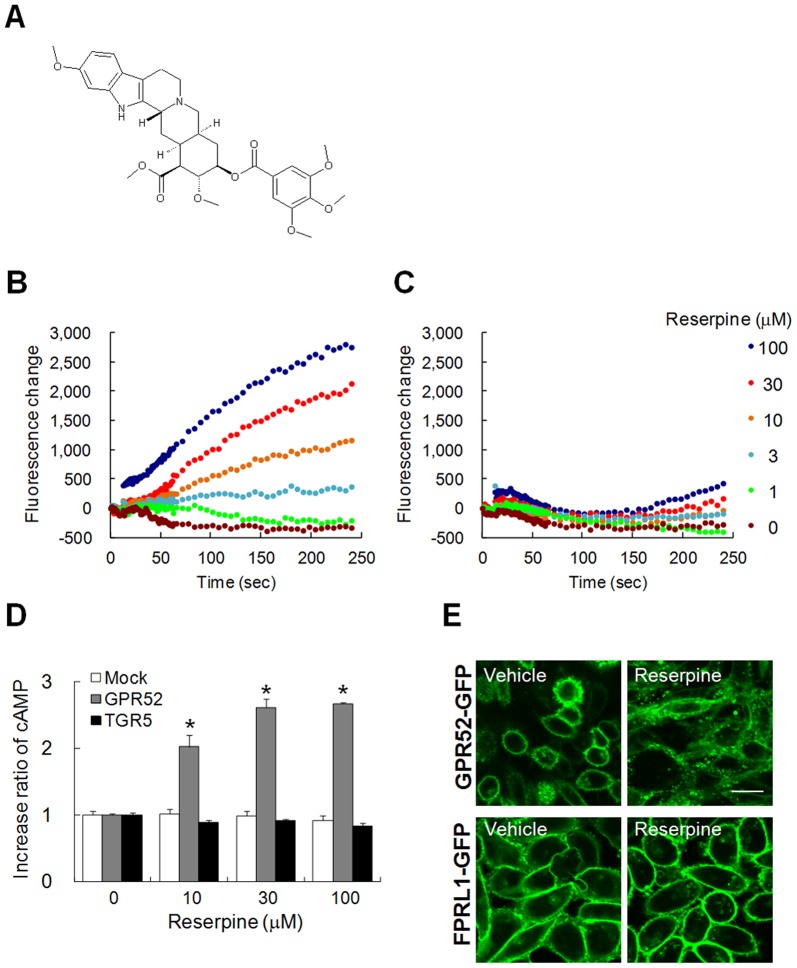
GPR52 is a Gs-coupled receptor activated by antipsychotics reserpine. **A,** The chemical structure of reserpine. **B–C,** Reserpine evoked calcium influx via GPR52 in HEK293 stably expressing mutated CNG channels (**B**). Calcium influx was elicited by reserpine from 1 to 100 µM when GPR52 was expressed. Meanwhile, Reserpine failed to exert calcium influx when the orphan GPCR, GPRC5D, was expressed (**C**). Representative dose-response curves were shown in **B** and **C**. **D,** GPR52-expressing CHO cells induced cAMP rise in a dose-dependent manner by reserpine from 10 to 100 µM whereas Mock- and TGR5-CHO cells failed to respond to reserpine. TGR5 is a Gs-coupled receptor[53]. Mean ± SEM (n = 4). *p<0.025, Williams test. **E,** Internalization of GPR52-GFP was induced by reserpine. GFP-fused GPR52 in CHO cells was stimulated by 30 µM recerpine for 30 min whereas GFP-fused FPRL1 did not. Bar: 20 µm.

To identify a ligand or an agonist for GPR88, GPR6, and GPR52, we performed *in vitro* high-throughput assays using mutated cyclic nucleotide-gated (CNG) cation channels gated by cAMP to screen a library of approximately 5,000 natural and synthetic compounds[19], [29]. We found that the antipsychotic drug reserpine selectively and dose-dependently activated GPR52 to evoke calcium influx (Figure 4A–C)[30]. We confirmed that reserpine elicited an increase in intracellular cAMP in GPR52-expressing CHO cells (Figure 4D) and specifically induced the internalization of GFP-fused GPR52 (Figure 4E), indicating that GPR52 is a druggable Gs-coupled receptor. Reserpine, isolated from the dried root of *Rauwolfia serpentina* (Indian snakeroot)[31], is an indole alkaloid antipsychotic drug that has been used for the relief of schizophrenic symptoms. Reserpine is rarely used today because of the development of better drugs and its numerous side-effects such as extrapyramidal symptoms (EPS) [32]. Although reserpine has been believed to ameliorate symptoms of schizophrenia by inhibiting the vesicular monoamine transporter[33], our findings raise the possibility that reserpine may also exert antipsychotic action via GPR52.

### Extensive analysis of gene expression and protein localization of GPR52

**Figure 5:**
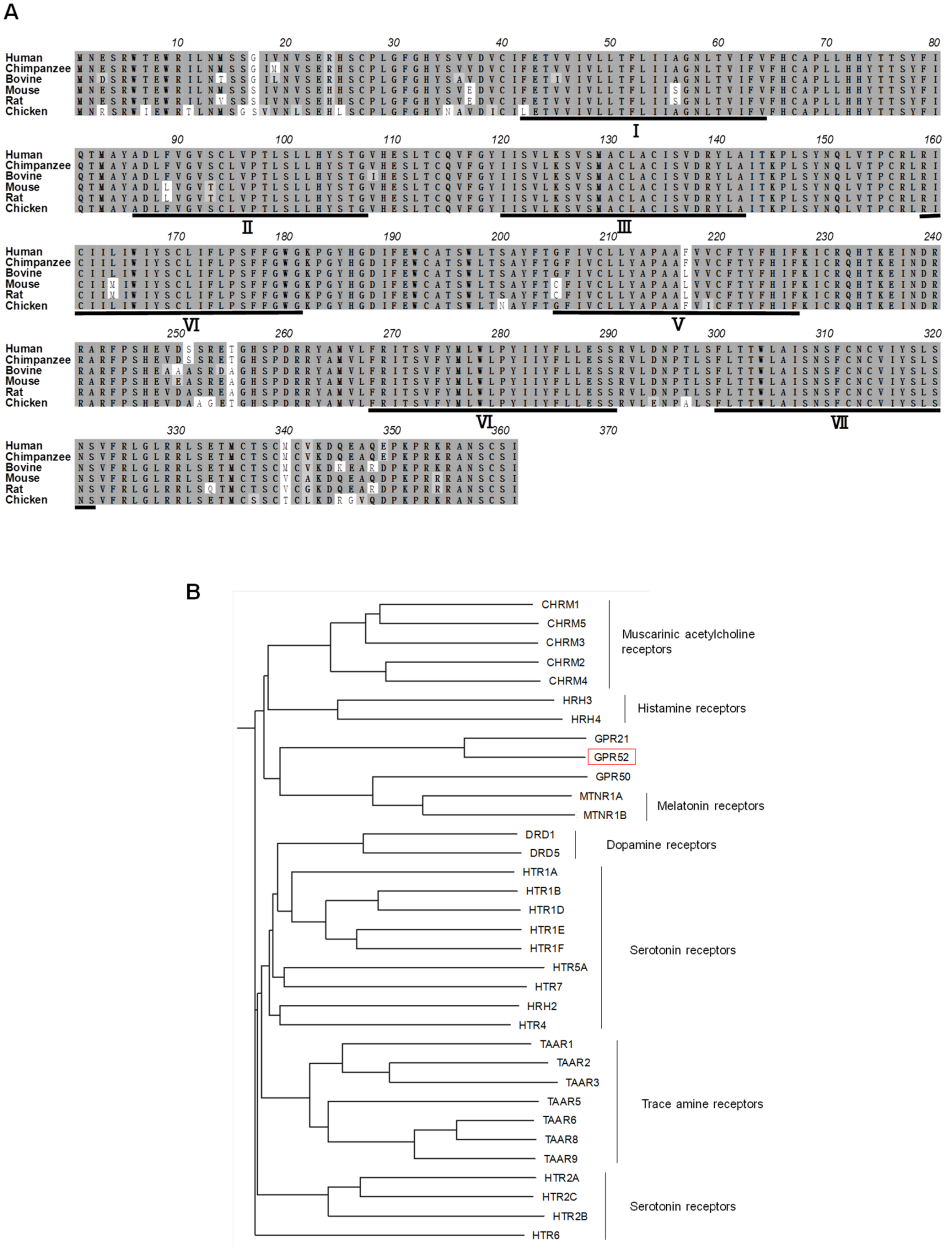
GPR52 is well conserved among vertebrates. **A,** Alignments of GPR52 amino acid sequences. Human GPR52 shares 99.7%, 95.8%, 95.2%, 94.7% and 91.9% identities with its chimpanzee, bovine, mouse, rat and chicken orthologues. **B,** Phylogenic tree showing protein sequence relationship of the human GPR52 and other GPCRs. CHRM: Muscarinic acetylcholine receptor families. HRH: Histamine receptor families. MTNR: Melatonin receptor families. DRD: Dopamine receptor families. HTR: 5-hydroxytryptamine receptor families. TAAR: Trace amine-associated receptor families.

**Figure 6:**
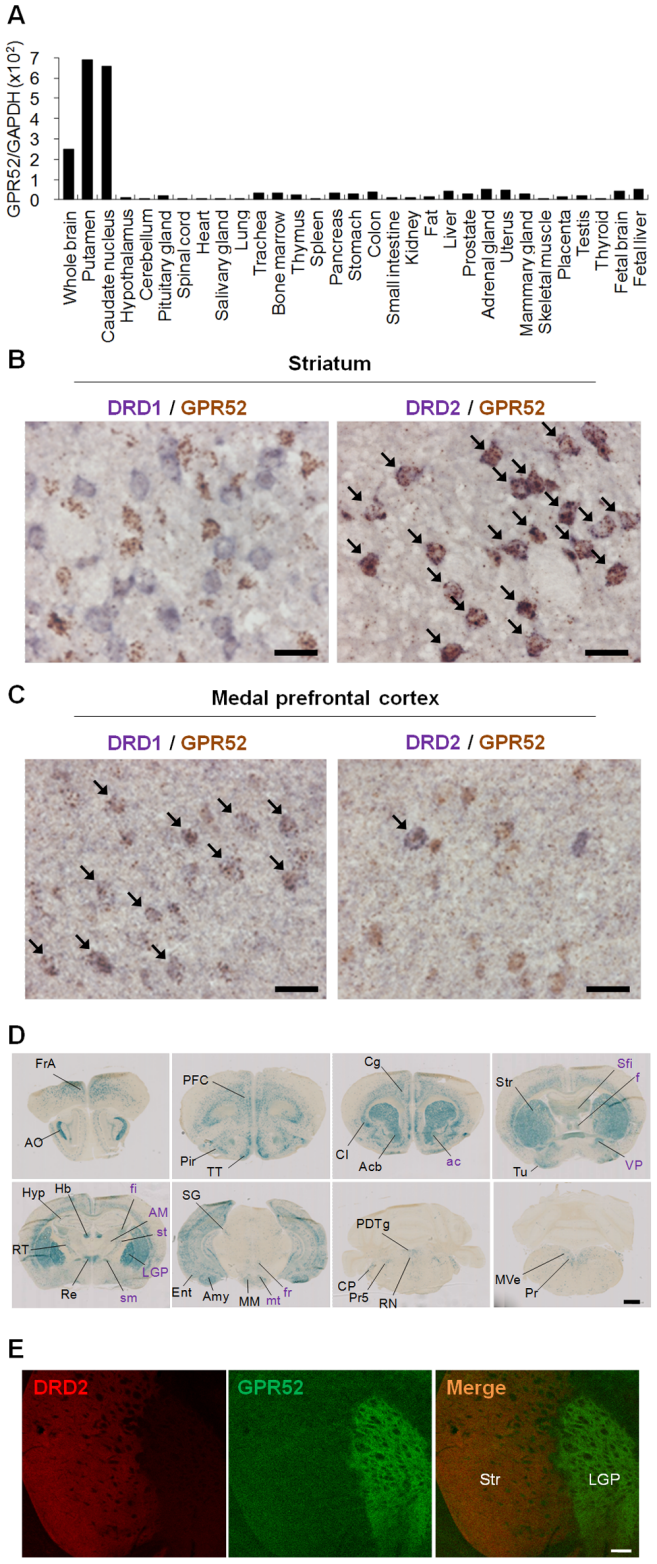
Gene expression distribution and protein localization of GPR52. **A,** GPR52 mRNA is abundantly expressed in human brain. GPR52 mRNAs were quantified by qPCR throughout human tissues. Data represent the ratios of GPR52 to glyceraldehydes-3-phosphate dehydrogenase (GAPDH) mRNA. **B–C,** ISH investigation of GPR52 mRNA was performed in rat striatum and medial prefrontal cortex. Double-ISH analysis of GPR52 with DRD1/DRD2 in adult male rats. Localizations of GPR52 and DRD1/DRD2 mRNAs were shown as brown and blue signals, respectively, in striatum (**B**) and medial prefrontal cortex (**C**). Allow indicates the double-stained neurons. Bar: 25 µm. **D,** Distribution of LacZ signals in GPR52-LacZ Tg mouse brain. Serial frontal brain sections (rostral → caudal) were stained with X-Gal in GPR52-LacZ Tg mouse. Black characters on the left side of the pictures show LacZ-positive cell bodies and fibers while purple characters on the right side show the fibers. Results and abbreviations were summarized in Table 2. Bar: 1 mm. **E,** Double detections of GFP signals with DRD2 in frontal brain sections of hGPR52-GFP Tg mouse. Green and red colors show GFP and DRD2 immunopositive signals, respectively. Bar: 200 µm.

GPR52 is highly conserved among vertebrates with over 90% of amino acid sequence identity (Figure 5A) and belongs to the GPCR family whose ligands are mainly small molecules, including neurotransmitters such as acetylcholine and dopamine (Figure 5B), suggesting that an endogenous ligand for GPR52 may be a small molecule. Tissue distribution analysis of human, mouse, and rat indicated that GPR52 is expressed exclusively in the brain, especially in striatum, with no significant differences amongst species (Figures 6A and S2A–C), suggesting that GPR52 possesses common functions beyond species. In human brain, the GPR52 expression profile overlapped with the distribution of D1 and D2 receptors (Figure S2D). Next, we conducted ISH studies of adult rat brain in detail and revealed that GPR52 was expressed in neurons in a variety of regions, including the medial prefrontal cortex, basolateral amygdaloid, and habenular nuclei (Figure S3 and Table 1), which are responsible for manifestations of psychiatric diseases[34]–[36]. Most intriguingly, GPR52 was largely co-localized with the D1 receptor in the medial prefrontal cortex, but co-localized with the D2 receptor in the basal ganglia (Figure 6B–C and 3D–F), suggesting that GPR52 may be involved in dopaminergic transmission at D1 receptor-expressing neurons in cortex and D2 receptor-expressing neurons in striatum. In schizophrenia, the D1 receptor is reduced in the prefrontal cortex in correlation with the severity of the negative symptoms and cognitive deficits[37], whereas D2 receptor signaling is considered to be overactivated[38]. Thus, our observation suggests that GPR52 may participate in the manifestation of schizophrenia.

**Table 1:** Distribution of GPR52 mRNA in the rat central nervous system.

		GPR52 mRNA
Telencephalon	frontal association cortex	++
	prelimbic cortex	++
	infralimbic cortex	++
	dorsal peduncular cortex	++
	ventral orbital cortex	+
	lateral orbital cortex	+
	dorsolateral orbital cortex	+
	piriform cortex	++
	cingulate cortex	++
	retrosplenial agranular cortex	+
	motor cortex	+
	somatosensory cortex	+
	granular insular cortex	+
	dysgranular insular cortex	+
	agranular insular cortex	+
	piriform cortex	+
	perirhinal cortex	+
	ectorhinal cortex	+
	lateral entorhinal cortex	++
	medial entorhinal cortex	++
	visual cortex	+
	primary auditory cortex	+
	anterior olfactory nucleus, medial part	+
	anterior olfactory nucleus, posterior part	+
	anterior olfactory nucleus, ventral part	+
	dorsal tenia tecta	++
	ventral tenia tecta	++
	ventral pallidum	+
	caudate putamen (striatum)	+++
	accumbens nucleus, core	+++
	accumbens nucleus, shell	+++
	olfactory tubercle	+++
	interstitial nucleus of the posterior limb of the anterior commissure	++
	bed nucleus of the stria terminalis	++
	lateral amygdaloid nucleus, dorsolateral part	+
	lateral amygdaloid nucleus, ventrolateral part	+
	lateral amygdaloid nucleus, ventromedial part	+
	basolateral amygdaloid nucleus, posterior part	+
	basolateral amygdaloid nucleus, anterior part	+
	medial amygdaloid nucleus, posterodorsal part	+
	basomedial amygdaloid nucleus, posterior part	+
	amygdalopiriform transition area	+
	posterolateral cortical amygdaloid nucleus	+
	posteromedial cortical amygdaloid nucleus	+
	amygdalohippocampal area, anterolateral part	+
	amygdalohippocampal area, posteromedial part	+
	granular layer of the dentate gyrus	+
	CA1-CA3 of pyramidal cell layer of the hippocampus	+
	subiculum	+
Diencephalon	anterior hypothalamic area	+
	posterior hypothalamic area	+
	lateral hypothalamic area	+
	arcuate hypothalamic nucleus, medial posterior part	+
	premammillary nucleus, ventral part	+
	premammillary nucleus, dorsal part	+
	medial mammillary nucleus, medial part	++
	lateral habenular nucleus	++
	medial habenular nucleus	+++
	paraventricular thalamic nucleus	+
	intermediodorsal thalamic nucleus	+
	central medial thalamic nucleus	+
	rhomboid thalamic nucleus	+
	reuniens thalamic nucleus	++
	reticular thalamic nucleus	+
	nucleus of the posterior commissure	+
Mesencephalon	substantia nigra, compact part	+
	substantia nigra, reticular part	+
	periaqueductal gray	+
	A8 dopamine cells	+
	interpeduncular nucleus	+

+++, highest; ++, moderate density; +, low density;

To address GPR52 receptor function, detailed gene expression, neuronal circuits and protein localization, we generated GPR52 knockout (KO) mice andthree types of transgenic mice in which LacZ, hGPR52, or hGPR52-GFP was overexpressed (under the control of the mouse GPR52 promoter of the BAC clone) that were termed GPR52-LacZ Tg, hGPR52 Tg, and hGPR52-GFP Tg mice, respectively (Figure S4). In the process of generating these transgenic mice, we first needed to generate GPR52 promoter-driven Cre (mGPR52-*Cre*) Tg mice that expressed the Cre under the control of mouse GPR52 promoter the BAC clone (Figure S4A). We first selected a single transgenic line, termed 116F, among several Tg mice in terms of appropriate Cre expression reflecting endogenous GPR52 distribution throughout tissues (Figure S4A). qPCR analysis showed that 116F expressed high levels of Cre mRNA in the brain whereas its low levels in peripheral tissues (Figure S5A). ISH study confirmed that the distribution of the Cre mRNA was apparently indistinguishable from that of endogenous GPR52 mRNA (Figure S5B), and the number of Cre-positive cells in 116F was equivalent to that of endogenous GPR52-positive cells in striatum as well as nucleus accumbens (Figure S5C-D and data not shown). To exclude any possibility of the ectopic expression of the Cre mRNA, we should directly examine the colocalization of Cre and GPR52 mRNAs. However, the double ISH study of the colocalization of Cre and GPR52 mRNAs was technically difficult. Alternatively, we tried to confirm the precise expression of the Cre mRNA by the double ISH staining using D1 and D2 receptor mRNAs because in the striatum almost all of the D2 receptor-expressing neurons expressed GPR52 mRNA (97%±2.1, Mean ± SEM) whereas all of the D1 receptor-expressing neurons did not expressed GPR52 mRNA at all, as shown in Figure 6B. Then, we confirmed that the Cre-positive cells of 116F were coexpressed with the D2 receptors but the D1 receptors in the striatum (Figure S5E-F). In addition, we have not observed any ectopic expressions of the Cre mRNA in 116F (Figure S5B and C). All taken together, we comprehensively concluded that this Cre expression reflects endogenous GPR52 expression pattern. Therefore, we used 116F as a most appropriate mGPR52-*Cre* Tg mice to generate three types of the transgenic mice for the following experiments.

To gain insight into neuronal circuits in which GPR52 is involved, we visualized axonal projections of neurons expressing GPR52 by using GPR52-LacZ Tg mice. GPR52-LacZ Tg mice showed LacZ expressions in cell bodies and fibers in the brain (Figures 6D and S6). These data are summarized in Table 2. The distribution of the LacZ-expressing cell bodies was almost identical to endogenous GPR52 mRNA localization level without the ectopic expression as shown in Table 2, supporting that the LacZ expression pattern reflects endogenous GPR52 expression. In agreement with that, the strong LacZ staining in the Tg mice were observed in the lateral globus pallidus (LGP) to which the D2 receptor/GPR52-expressing MSNs are known to be directly projected[5] (Figure 6D and S6A). This result is consistent with the GPR52-GFP localization in the LGP in Figure 6E. Although a LacZ may not be the most effective indicator of axonal projections[39], we were able to observe the distinguishable projections stained with LacZ, as shown in the LGP. Numerous papers also have reported that LacZ staining in the transgenic mice can recognize axonal projections[40]–[42]. Therefore, we conclude that our LacZ stains can detect axonal projections. Interestingly, we clearly recognized the LacZ-stained fibers in the mammilothalamic tract that is known to be projected from the mammillary nucleus to the anterior thalamic neucleus[43]. In addition, we found that LacZ expression was also detected in the fornix projected from the hippocumpus probably to the mammillary nucleus (Figure S6B). It is reported that these projections were included in a limbic neural circuit that are important for recollection memory, especially spatial memory[43]. We also observed that the LacZ-positive neurons apparently projected to the midbrain from the habenular nucleus (Figure S6C), in which a negative reward signal in dopaminergic neurons originates[44].

**Table 2:** Distributions of mGPR52 mRNA in wild type mice and LacZ signals in mGPR52-LacZ Tg mice.

		Wild type mice	mGPR52-LacZ Tg mice	
Abbreviation	Structure name	mGPR52 mRNA	Cell body with fiber	Fiber only
ac	anterior commissure			○
Acb	accumbense nucleus	+++	○	
AM	anteromedial thalamic nucleus			
Amy	amygdaloid nucleus	++	○	
AO	anterior olfactory nucleus	+	○	
AV	anteroventral thalamic nucleus			○
Cg	cingulate cortex	+	○	
Cl	claustrum	+	○	
CP	cochlear nucleus	+	○	
CP4V	Choloid plexus of 4v		○	
Cu	cuneate fasciculus	+	○	
DMTg	dorsomedial tegmental area			○
Ent	Entorhinal cortex	+	○	
f	fornix			○
fi	fimbria of the hippocampus			○
fr	fasciculus retroflexus			○
FrA	frontal association cortex	+	○	
LGP	lateral globus pallidus			○
Hb	habenular nucleus	+++	○	
hbc	habenular commissure			○
Hyp	hippocampus	+	○	
IP	interpeduncular nucleus	+	○	
LH	lateral hypothalamic area	+	○	
mfb	medial forebrain bundle			○
MM	mammillary nucleus	+	○	
mt	mammillothalamic tract			○
Mve	medial vestibular nucleus	+	○	
PDTg	posterodorsal tegmental nucleus			○
PFC	prefrontal cortex	+	○	
Pir	piriform cortex	+	○	
Pr	prepositus nucleus	+	○	
Pr5	principal sensory trigeminal nucleus	+	○	
PRh	Perihinal cortex	+	○	
PT	pretectal nucleus	+	○	
Re	reuniens thalamic nucleus	+	○	
RI	rostral interstitial nucleus of medial longitudinal fasciculus	+	○	
RN	raphe nucleus	+	○	
RS	retrosplenial cortex	+	○	
RT	reticular thalamic nucleus	+	○	
Sfi	septofimbrial nucleus			○
SG	suprageniculate thalamic nucleus	+	○	
sm	stria medullaris of the thalamus			○
st	stria terminalis			○
Str	striatum	+++	○	
TS	triangular septal nucleus	+	○	
TT	tenia tacta	+	○	
Tu	Olfactory tubecle	+	○	
Ve	vestibular nucleus			○
VP	ventral pallidum			○
ZI	zona incerta	+	○	

mGPR52 mRNA.

+++, highest; ++, moderate density; +, low density;

mGPR52-LacZ Tg mice.

○, LacZ-positive.

In order to characterize cortical GPR52-expressing neurons, we performed a double ISH analysis in hGPR52 Tg mice of hGPR52 together with VGluT1 (vesicular glutamate transporter 1), NR1 (N-methyl-D-aspartate (NMDA) receptor subunit NR1), GAD67 (glutamic acid decarboxylase 67), or PV (parvalbumin) (Figure S7E-H), which were used as markers of glutamatergic, NMDAergic, and GABAergic neurons, respectively[45], [46]. Our data indicated that almost all of the GPR52-expressing neurons in the prefrontal cortex are glutamatergic whereas only about 10% of the neurons are GABAergic.

GPCRs are well-known to be expressed on dendrites and/or axons, stay in pre- and/or post-synaptic sites and mediate slow synaptic transmission, leading to long-lasting forms of synaptic plasticity[3]. We have thus far failed to obtain a reliable antibody against the receptor. We have therefore attempted to elucidate GPR52 localization using hGPR52-GFP Tg mice in which the GFP (green fluorescent protein)-fused hGPR52 was functional *in vitro* (data not shown). hGPR52-GFP Tg mice exhibited green fluorescence throughout the brain (Figure S8) that reflects rat distribution of GPR52 mRNA or LacZ profiles of GPR52-LacZ Tg mice as described above. Interestingly, hGPR52-GFP and D2 receptor protein l were clearly divided around striatal regions. GPR52-GFP was mainly seen in the lateral globus pallidus (LGP) whereas most of the D2 receptor protein remained localized in striatum (Figure 6E). This result suggests that GPR52 in striatal neurons is actively transported to axon terminals near LGP while the D2 receptor is dendritic because mRNAs of both GPR52 and D2 receptor are abundantly expressed in same striatal neurons.

### Phenotypes of hGPR52 Tg and GPR52 KO mice

**Figure 7:**
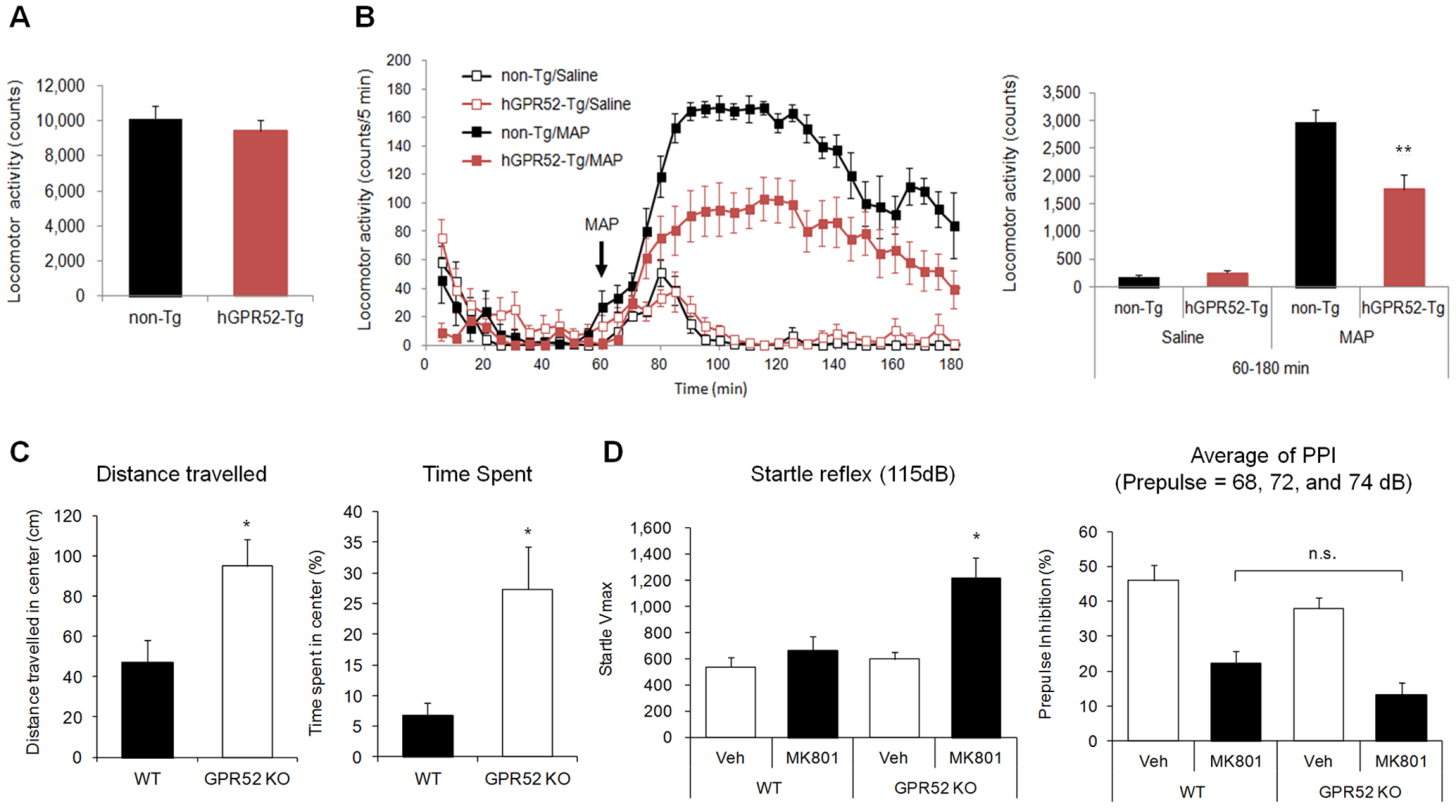
Behavioral characterizations of hGPR52 Tg and GPR52 KO mice. **A,** Spontaneous locomotor activities of hGPR52 Tg and non-Tg mice for 16 hours were monitored under the controlled condition of light. Animals were exposed to light for 12 hours during the experiment. **B,** Effects of MAP on locomotor activity of hGPR52 Tg and non-Tg mice were shown. Mean±SEM (n = 7–10). **p<0.01, Student's t-test. **C-D,** Exploratory activity (left) and time spent (right) in the open-field test over 5min (**C**), and startle reflex (left) and prepulse inhibition of the startle reflex (right) (**D**) in GPR52 KO and WT mice. Mean±SEM (n = 10). *p<0.05, **p<0.01, Student's t-test.

To elucidate the function of GPR52, we performed a phenotypic analysis of hGPR52 Tg and GPR52 KO mice (Figure 7). Using hGPR52 cRNA probe that can also detect endogenous mGPR52 mRNA because of the highly conserved nucleic acid sequence identity with over 90%, ISH analysis showed that the signals were markedly more increased in hGPR52-Tg mice than in non-Tg mice (Figure S7A–C). We have not detected any obvious differences in the expression pattern of the signals between hGPR52-Tg and non-Tg mice. We also observed that hGPR52 mRNAs were robustly expressed in hGPR52-Tg mice by qPCR (Figure S7D). These results suggested that hGPR52 mRNA was overexpressd at appropriate cells in hGPR52-Tg mice instead of the ectopic overexpression. Thus, we assume that GPR52-mediated signal pathway was more activated in hGPR52-Tg mice. Both hGPR52 Tg and GPR52 KO mice were normal in body and brain weight (data not shown). hGPR52 Tg mice showed normal locomotor activity under normal conditions (Figure 7A), whereas they were observed to have attenuated methamphetamine (MAP)-induced hyperlocomotion as compared with that of non-Tg mice (Figure 7B). There was no significant difference in MAP-induced hyperlocomotion between GPR52 KO and wild type (WT) mice (data not shown). This result suggested that overexpression of hGPR52 may counteract hyperdopaminergic transmission by MAP, a psychostimulant excessively releasing dopamine in the brain[47]. To demonstrate this hypothesis, we need to investigate GPR52-mediated signaling, transmission, and behavior pharmacologically, using a GPR52 agonistic compound.

In the open field test, GPR52 KO mice stayed and moved around the central zone significantly longer than WT littermates (Figure 7C). No difference between genotypes was observed in the total distance traveled (data not shown) and brain morphology (Figure S9). Thus, GPR52 KO mice displayed anxiolytic-like behavior[48]. Since GPR52 mRNA is expressed in the limbic system which is tightly linked with emotion and anxiety [49], our finding raised the possibility that GPR52 hypofunction may exert anxiolytic action. Further investigation is required to reveal the mechanism leading to this anxiolytic-like behavior in GPR52 KO mice.

Schizophrenic patients have impaired startle habituation and prepulse inhibition (PPI) of the startle reflex, typical and atypical antipsychotics have the common feature of diminishing startle response and ameliorating PPI deficits[50]. We next investigated sensorimotor gating of GPR52 KO mice using an NMDAR antagonist MK-801, which is used to mimic psychosis of schizophrenia[51]. There was no difference between WT and GPR52 KO mice either in the level of PPI or the degree of defict evoked by MK801(Figure 7D). However, GPR52 KO mice were much more sensitive to the startle response following administration of MK-801 (Figure 7D). Taken together with our observations, we proposed that GPR52 can modulate dopamine and NMDA systems, dysfunctions of which have been proposed to cause symptoms of schizophrenia[47], [52]. Further studies are required to clarify the mechanisms involved in the modification by GPR52 of neurotransmission and neuronal signal transduction.

In summary, we show comprehensive gene expression profiles of 322 non-odorant GPCRs throughout mouse tissues and disclose a number of GPCRs that are exclusively expressed in the CNS, especially in the striatum where D1 and D2 receptors are most abundant. Moreover, we show that among them, GPR52 may be a promising therapeutic target for psychiatric disorders. Our approach may expedite the identification of other potential therapeutic targets and will help predict their functions and side effects *in vivo*.

## Supporting Information

Figure S1 **Clustering for expression profile of GPCR gene. A,** Heatmap of expression profile of GPCR gene throughout mouse tissues by qPCR. GPCRs and tissues are aligned vertically and horizontally, respectively. Using Euclidean-ward methods, 322 GPCRs were divided into 14 clusters while CNS tissues were obviously distinguishable as a cluster from peripheral tissues. Yellow-black color scale indicates mRNA copy numbers per 25 ng total RNA as log 2 ratios (Tables S1 and S2). (**B**) represents box plots of log 10 ratios of averaged mRNA expression level of CNS over that of peripheral tissues for each GPCR. Cluster 1, 3, 4, 11, 12, and 14 exhibited relatively abundant expressions in CNS. Cluster 2 was not enriched in CNS because of its low expression in CNS according to (**A**).
(TIF)

Figure S2 **Gene expressions of GPR52 throughout the tissues in human, mice, and rat.** **A-C,** quantitative real-time PCR of GPR52 mRNA in human (**A**), mouse (**B**), and rat tissues (**C**). **D,** Distribution of GPR52, DRD1, DRD2, and mGluR5 in human brain regions. Data represent the ratios of GPR52 to glyceraldehydes-3-phosphate dehydrogenase (GAPDH) mRNA. Each column represents a mean value in duplicate determinations.
(TIF)

Figure S3 **ISH investigation of GPR52 mRNA in rat and mouse CNS.** **A–F,** Expression of GPR52 in adult male rats revealed by ISH. Anti-sense probe signals were detected in medial prefrontal cortex (**A**), striatum (**B**), accumbens nucleus (**C**), basolateral amygdaloid nucleus (**D**), habenular nucleus (**E**), and mammillary nucleus (**F**). Results were summarized in Table 1. **G–L,** Expression of GPR52 in adult male mice revealed by ISH. Anti-sense probe signals were detected in medial prefrontal cortex (**G**), piriform cortex (**H**), striatum (**I**), accumbens nucleus (**J**), basolateral amygdaloid nucleus (**K**), habenular nucleus, and hippocampus (**L**). Results were summarized in Table 2. Abbreviations were shown in Table 2. Bar: 0.2 mm.
(TIF)

Figure S4 **Construction map of three types of transgenic mice.** **A,** By being crossed with Cre-expressing transgenic mice, termed mGPR52-*Cre* Tg mice, with artificial chromosome (BAC) carrying Cre driven by mouse GPR52 (mGPR52) promoter, hGPR52 Tg, hGPR52-GFP Tg, and GPR52-LacZ Tg mice were generated to overexpress ubiquitin (Ubc) promoter-driven human GPR52, GFP-fused human GPR52, and ROSA26 promoter-driven LacZ reporter, respectively. **B,** Gene targeting strategy to generate GPR52 KO mice (Table S5). **C,** Genotyping of GPR52 KO mice by PCR amplification.
(TIF)

Figure S5 **Expression pattern of Cre mRNA in mGPR52-** ***Cre*** **Tg mice, 116F.** **A,** the Cre expression level of various tissues in 116F were examined by qPCR. **B,** Distributions of Cre mRNA and endogenous GPR52 mRNA in basal ganglions in 116F detected by ISH. Bar: 1 mm. **C–D,** Cre mRNA positive neurons in striatum were examined by ISH (**C**). Bar: 100 µm. Numbers of Cre and GPR52 mRNA positive neurons were counted in 116F (**D**). Mean ± SEM (n = 3). **E–F,** Double-ISH study of Cre mRNA and D1R (**E**) or D2R (**F**) mRNA in 116F. Red and green signals show Cre mRNA and D1R/D2R mRNA, respectively. Arrows show double positive neurons. Bar: 20 µm.
(TIF)

Figure S6 **LacZ expressions in brain of GPR52-LacZ Tg mice.** **A,** Serial frontal brain sections stained with X-Gal in the GPR52-LacZ Tg mouse (rostral → caudal). Black characters on the left side of the pictures show the area existing the LacZ-positive cell bodies and fibers, and purple characters on the right side show the area existing only the LacZ-positive fibers. Results and abbreviations were summarized in Table 2. Bar: 1 mm. **B,** Projections of the LacZ-positive neurons were observed from mammillary nucleus and hippocumpus. Sagital brain sections of the GPR52-LacZ Tg mice were stained with X-Gal. The red arrows show projections of the neurons expressing the LacZ signals in mammilothalamic tract and fornix. **C,** Projection of the LacZ-positive neurons from habenular nucleus to midbrain. Serial sagital brain sections showed that the LacZ-positive neurons in habenular nucleus project to substantia nigra and ventral tegmental area via fasciculus retroflexus. Bar: 0.5 mm.
(TIF)

Figure S7 **Characterization of GPR52-expressing neurons in hGPR52 Tg mice. A–C,** GPR52 mRNAs were detected by ISH using hGPR52 cRNA probe in prefrontal cortex (**A**), striatum (**B**), and hippocampus (**C**) in hGPR52 Tg mice. Bar: 0.25 mm (**A**), and 0.5 mm (**B** and **C**). Because the hGPR52 cRNA probe was crossreacted to endogenous GPR52 mRNA in the non-Tg mice, the signals were significantly more increased in hGPR52-Tg mice than in non-Tg mice. **D,** Gene expression levels of hGPR52 and endogenousGPR52 (mGPR52) in brain were examined by qPCR. mGPR52 and hGPR52 mRNA levels were normalized by cyclophilin. Mean ± SEM (n = 5). The hGPR52 mRNA was highly expressed in the hGPR52-Tg mice while the endogenous GPR52 (mGPR52) mRNA showed similar expression levels in hGPR52-Tg and non-Tg mice. **E–H,** Double-ISH investigation of GPR52 with VGluT1 (**E**), GAD67 (**F**), NR1 (**G**), and PV (**H**) in prefrontal cortex of hGPR52 Tg mice. Red shows GPR52 and green in each picture shows VGluT1, GAD67, NR1, and PV. Arrows show double positive neurons. In (**F**) and (**G**), almost all of the GPR52-expressing neurons expressed the VGluT1 and the NR1 mRNAs. Bar: 100 µm.
(TIF)

Figure S8 **GFP fluorescence in brain of hGPR52-GFP Tg mice.** **A–H,** GFP signals in coronal brain sections of hGPR52-GFP Tg mice were detected by confocal microscopy. All of the GFP signals were observed in fibers, but not in cell bodies. Abbreviations were shown in Table 2. Bar: 200 µm.
(TIF)

Figure S9 **Brain morphology of GPR52 KO mice. A,** Whole body and brain weights in GPR52 KO mice and WT littermates (5-month-old males). Mean±SEM (n = 8). **B–D,** Brain slices were stained using the NeuN antibody. **B,** Pictures show representative cortical sections. Scale bar, 100 µm. Bar graphs indicates widths of the layers (upper) and NeuN density (lower) in cortex. Mean±SEM (n = 3). **C,** Pictures show representative brain slices around nucleus accumbens and striatum. Scale bar, 400 µm. Bar graphs indicate NeuN density in those regions. Mean±SEM (n = 3). **D,** Pictures show representative brain slices of CA1 and dentate gyrus (DG) in hippocampus. Scale bar, 400 µm (left) and 50 µm (middle and right). Bar graphs indicate widths of pyramidal cell layer of CA1 and DG as well as NeuN density. Mean±SEM (n = 3).
(TIF)

Table S1 Data set of the comprehensive GPCR expression analysis.
(XLSX)

Table S2 TaqMan probe and primer sequences used for the comprehensive GPCR expression analysis.
(XLSX)

Table S3 Information of the examined GPCRs.
(XLSX)

Table S4 TaqMan probe and primer sequences.
(XLSX)

Table S5 PCR primers used for the generation of GPR52 KO mice.
(XLSX)
