# Supplementary figures and images for: Anatomical Transcriptome of G Protein-Coupled Receptors Leads to the Identification of a Novel Therapeutic Candidate GPR52 for Psychiatric Disorders
Source: PLoS One. 2014 Feb 28;9(2):e90134. doi: 10.1371/journal.pone.0090134 (PMC3938596; doi:10.1371/journal.pone.0090134)

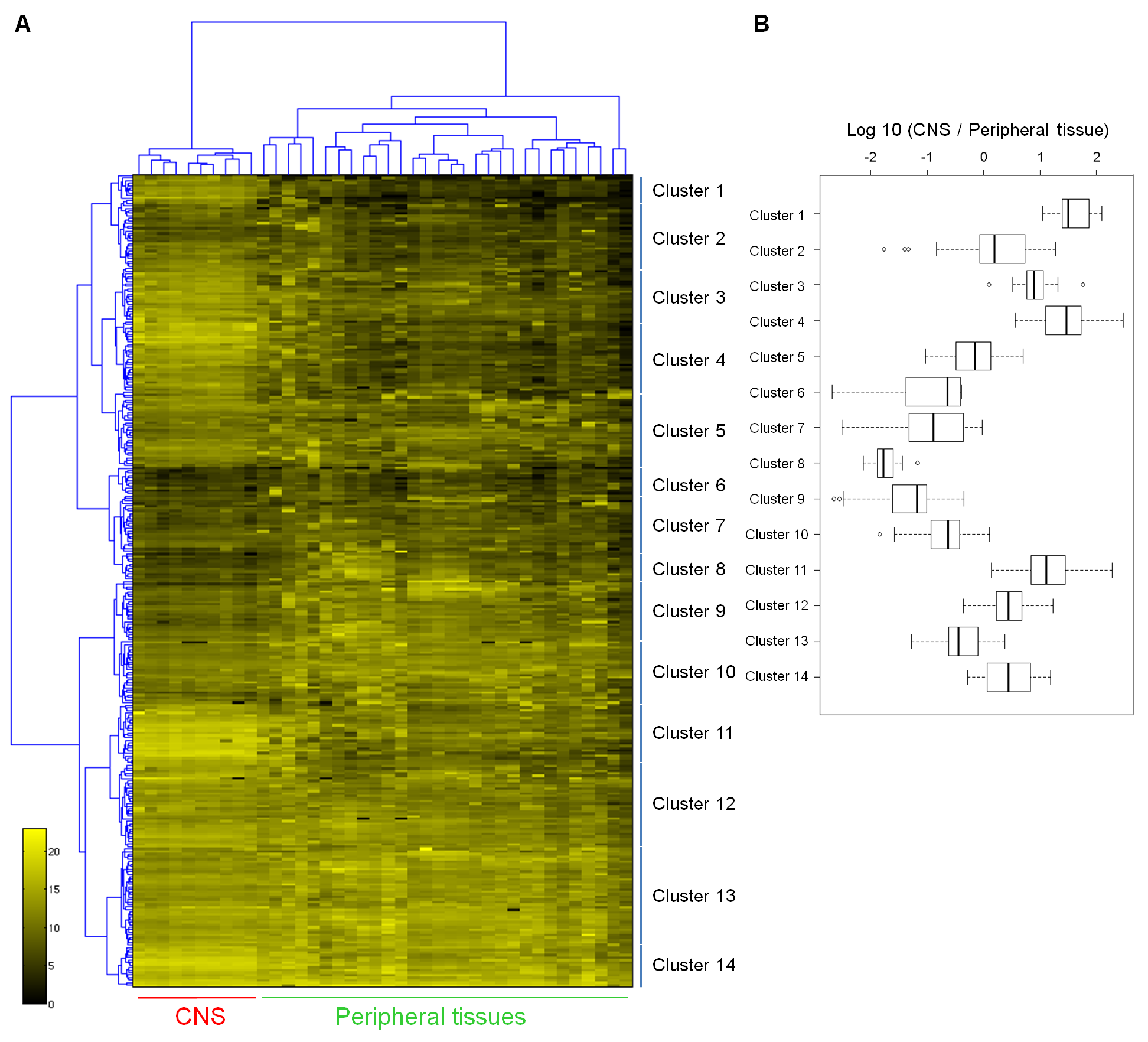

Supplement: Figure S1 — Clustering for expression profile of GPCR gene. A, Heatmap of expression profile of GPCR gene throughout mouse tissues by qPCR. GPCRs and tissues are aligned vertically and horizontally, respectively. Using Euclidean-ward methods, 322 GPCRs were divided into 14 clusters while CNS tissues were obviously distinguishable as a cluster from peripheral tissues. Yellow-black color scale indicates mRNA copy numbers per 25 ng total RNA as log 2 ratios (Tables S1 and S2). (B) represents box plots of log 10 ratios of averaged mRNA expression level of CNS over that of peripheral tissues for each GPCR. Cluster 1, 3, 4, 11, 12, and 14 exhibited relatively abundant expressions in CNS. Cluster 2 was not enriched in CNS because of its low expression in CNS according to (A). (TIF) [file pone.0090134.s001.tif]

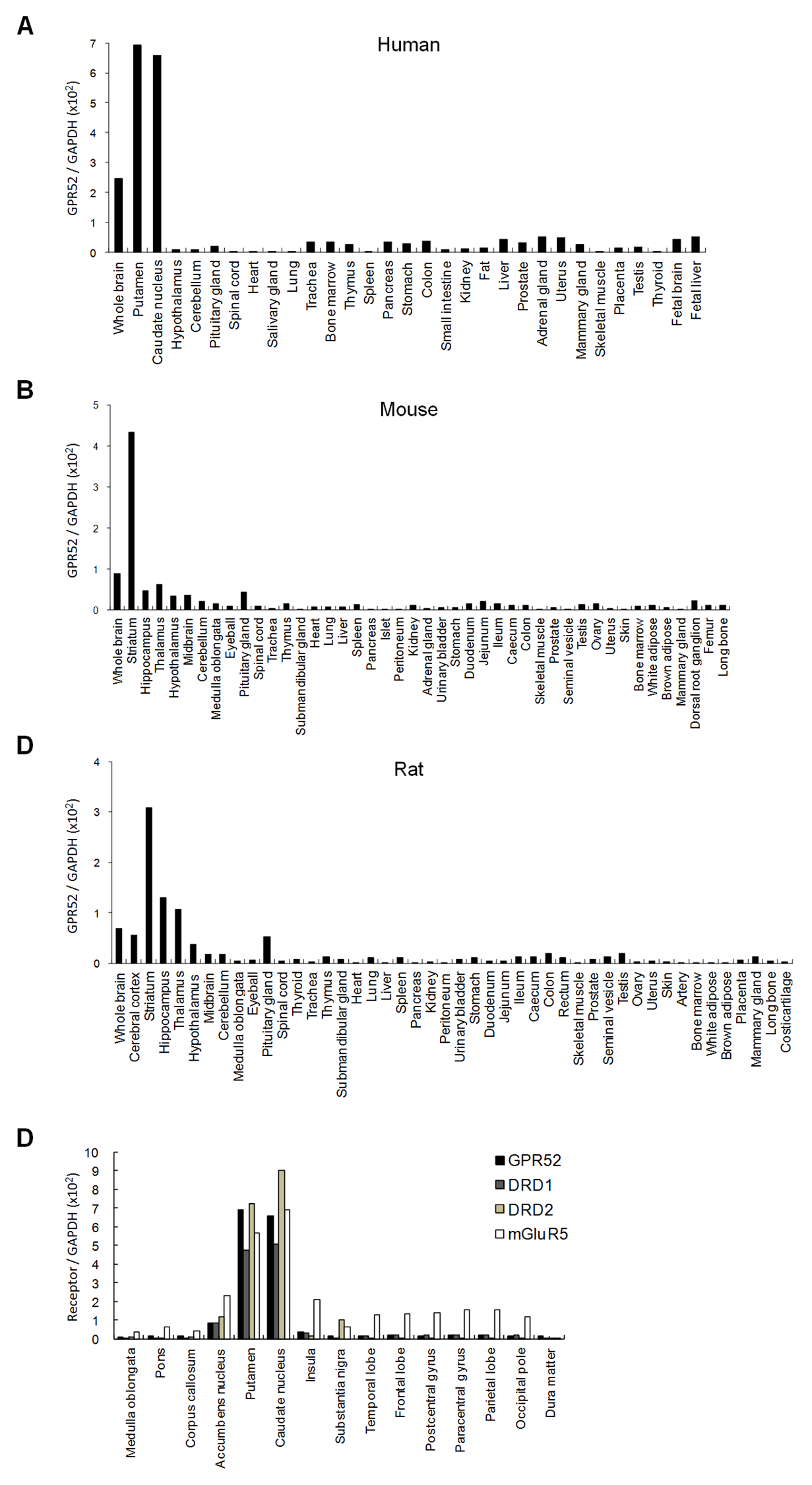

Supplement: Figure S2 — Gene expressions of GPR52 throughout the tissues in human, mice, and rat. A-C, quantitative real-time PCR of GPR52 mRNA in human (A), mouse (B), and rat tissues (C). D, Distribution of GPR52, DRD1, DRD2, and mGluR5 in human brain regions. Data represent the ratios of GPR52 to glyceraldehydes-3-phosphate dehydrogenase (GAPDH) mRNA. Each column represents a mean value in duplicate determinations. (TIF) [file pone.0090134.s002.tif]

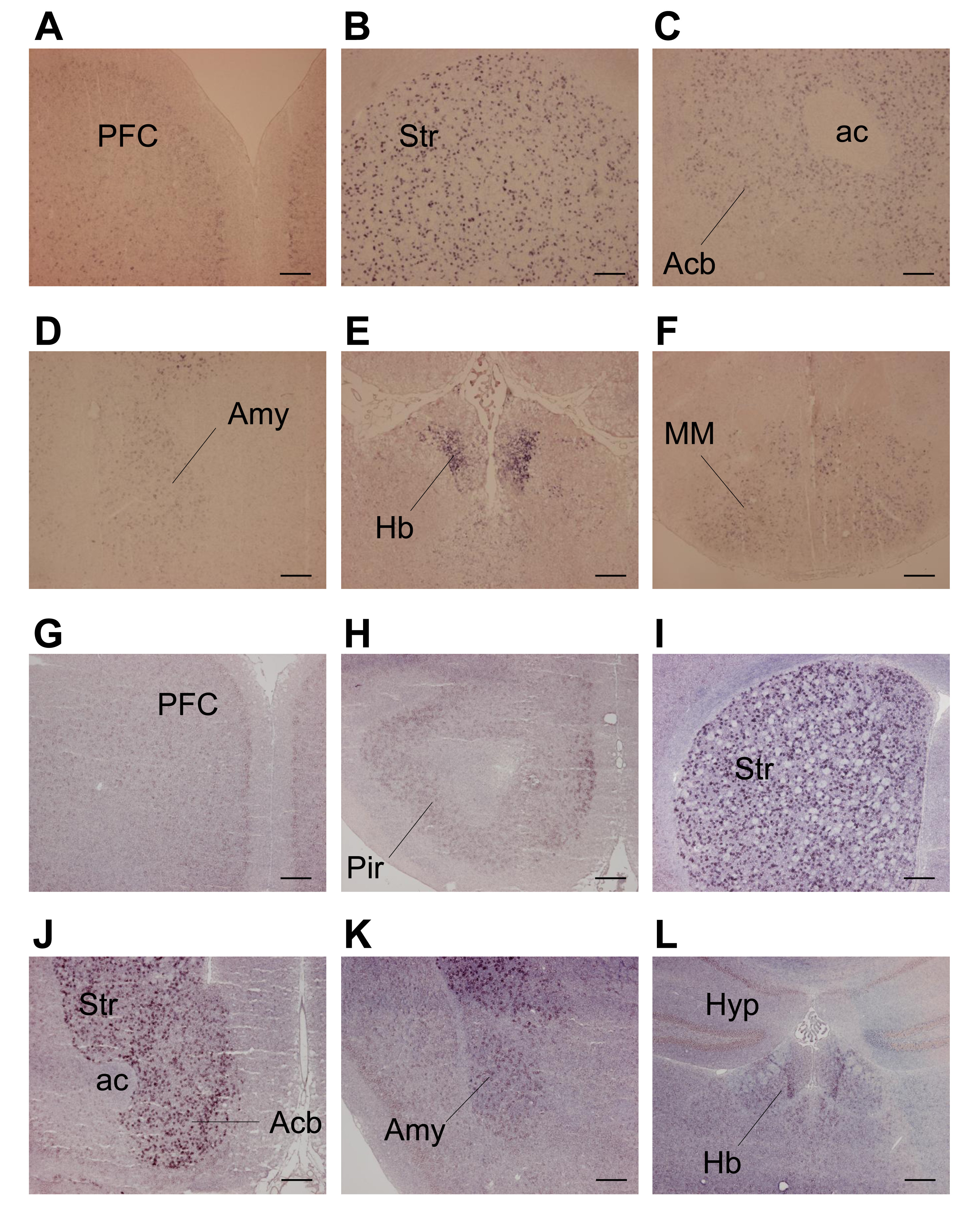

Supplement: Figure S3 — ISH investigation of GPR52 mRNA in rat and mouse CNS. A–F, Expression of GPR52 in adult male rats revealed by ISH. Anti-sense probe signals were detected in medial prefrontal cortex (A), striatum (B), accumbens nucleus (C), basolateral amygdaloid nucleus (D), habenular nucleus (E), and mammillary nucleus (F). Results were summarized in Table 1. G–L, Expression of GPR52 in adult male mice revealed by ISH. Anti-sense probe signals were detected in medial prefrontal cortex (G), piriform cortex (H), striatum (I), accumbens nucleus (J), basolateral amygdaloid nucleus (K), habenular nucleus, and hippocampus (L). Results were summarized in Table 2. Abbreviations were shown in Table 2. Bar: 0.2 mm. (TIF) [file pone.0090134.s003.tif]

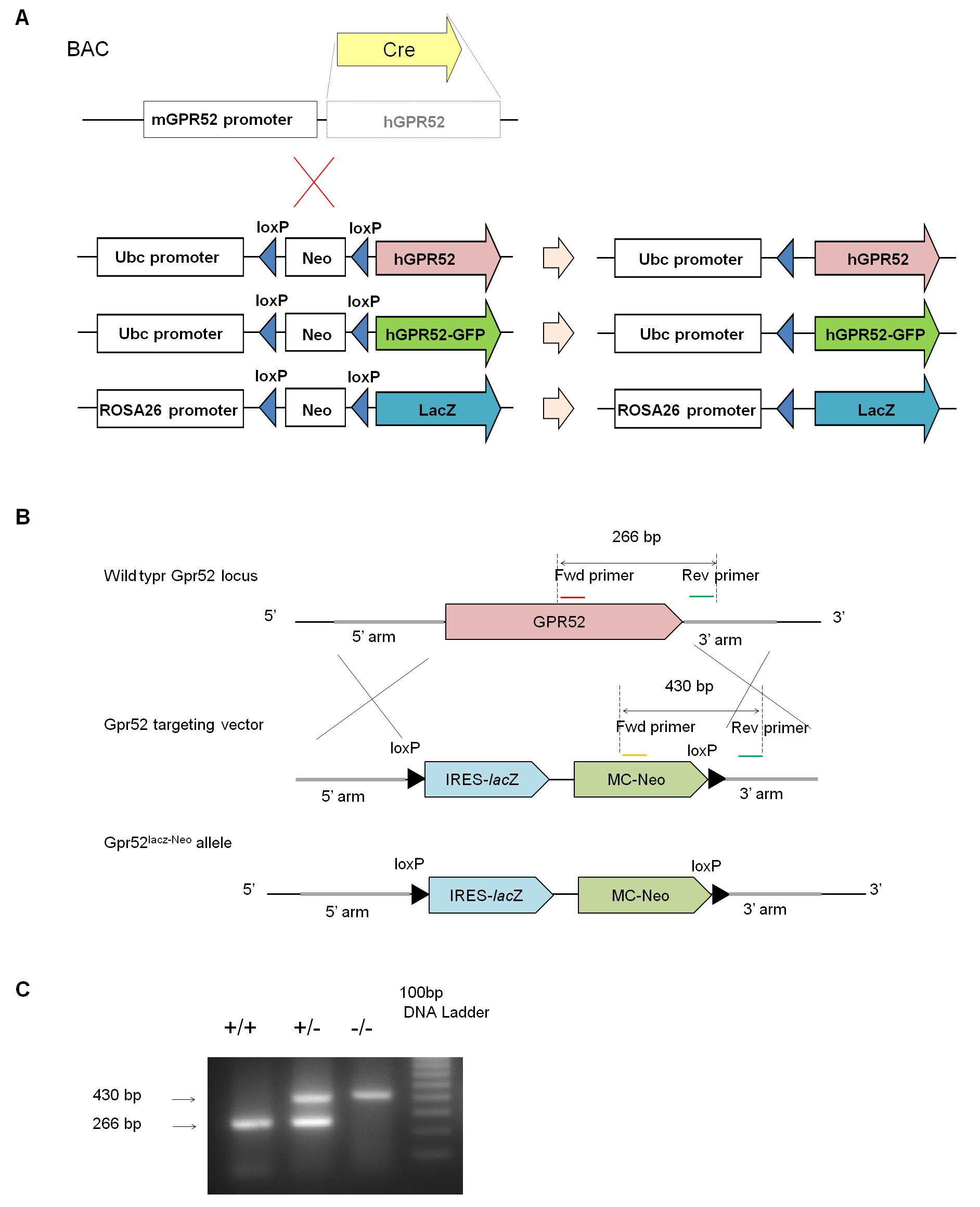

Supplement: Figure S4 — Construction map of three types of transgenic mice. A, By being crossed with Cre-expressing transgenic mice, termed mGPR52-Cre Tg mice, with artificial chromosome (BAC) carrying Cre driven by mouse GPR52 (mGPR52) promoter, hGPR52 Tg, hGPR52-GFP Tg, and GPR52-LacZ Tg mice were generated to overexpress ubiquitin (Ubc) promoter-driven human GPR52, GFP-fused human GPR52, and ROSA26 promoter-driven LacZ reporter, respectively. B, Gene targeting strategy to generate GPR52 KO mice (Table S5). C, Genotyping of GPR52 KO mice by PCR amplification. (TIF) [file pone.0090134.s004.tif]

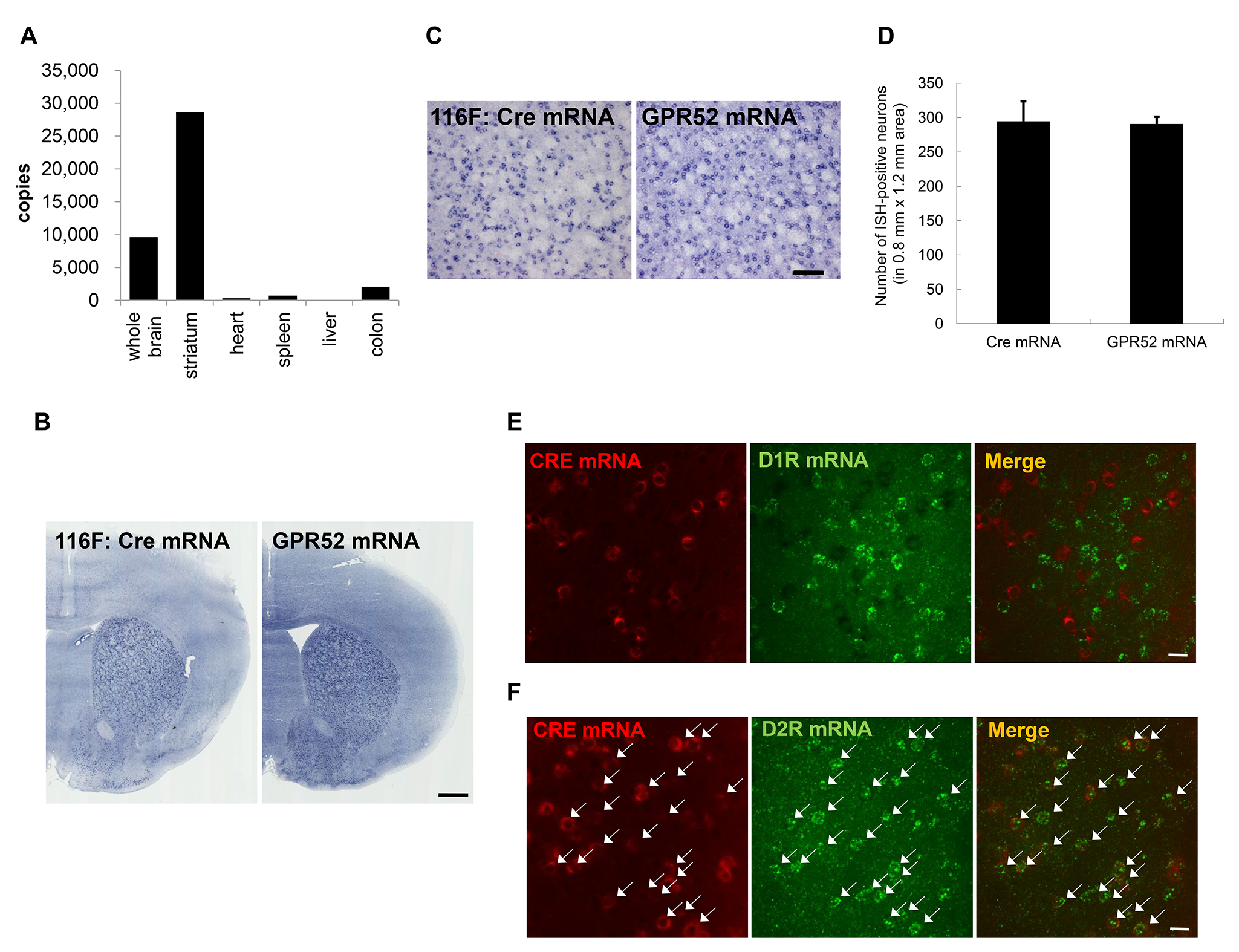

Supplement: Figure S5 — Expression pattern of Cre mRNA in mGPR52- Cre Tg mice, 116F. A, the Cre expression level of various tissues in 116F were examined by qPCR. B, Distributions of Cre mRNA and endogenous GPR52 mRNA in basal ganglions in 116F detected by ISH. Bar: 1 mm. C–D, Cre mRNA positive neurons in striatum were examined by ISH (C). Bar: 100 µm. Numbers of Cre and GPR52 mRNA positive neurons were counted in 116F (D). Mean ± SEM (n = 3). E–F, Double-ISH study of Cre mRNA and D1R (E) or D2R (F) mRNA in 116F. Red and green signals show Cre mRNA and D1R/D2R mRNA, respectively. Arrows show double positive neurons. Bar: 20 µm. (TIF) [file pone.0090134.s005.tif]

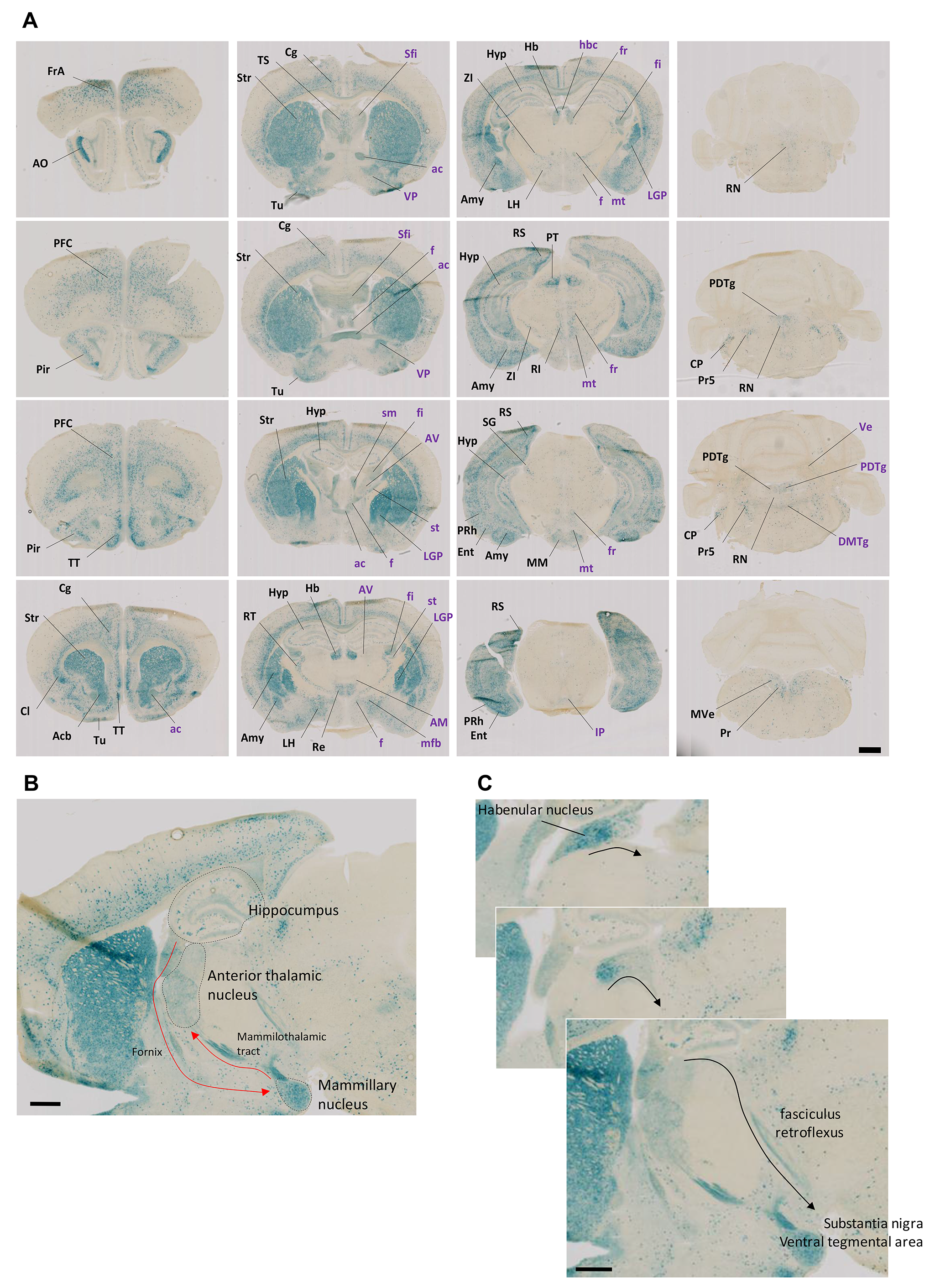

Supplement: Figure S6 — LacZ expressions in brain of GPR52-LacZ Tg mice. A, Serial frontal brain sections stained with X-Gal in the GPR52-LacZ Tg mouse (rostral → caudal). Black characters on the left side of the pictures show the area existing the LacZ-positive cell bodies and fibers, and purple characters on the right side show the area existing only the LacZ-positive fibers. Results and abbreviations were summarized in Table 2. Bar: 1 mm. B, Projections of the LacZ-positive neurons were observed from mammillary nucleus and hippocumpus. Sagital brain sections of the GPR52-LacZ Tg mice were stained with X-Gal. The red arrows show projections of the neurons expressing the LacZ signals in mammilothalamic tract and fornix. C, Projection of the LacZ-positive neurons from habenular nucleus to midbrain. Serial sagital brain sections showed that the LacZ-positive neurons in habenular nucleus project to substantia nigra and ventral tegmental area via fasciculus retroflexus. Bar: 0.5 mm. (TIF) [file pone.0090134.s006.tif]

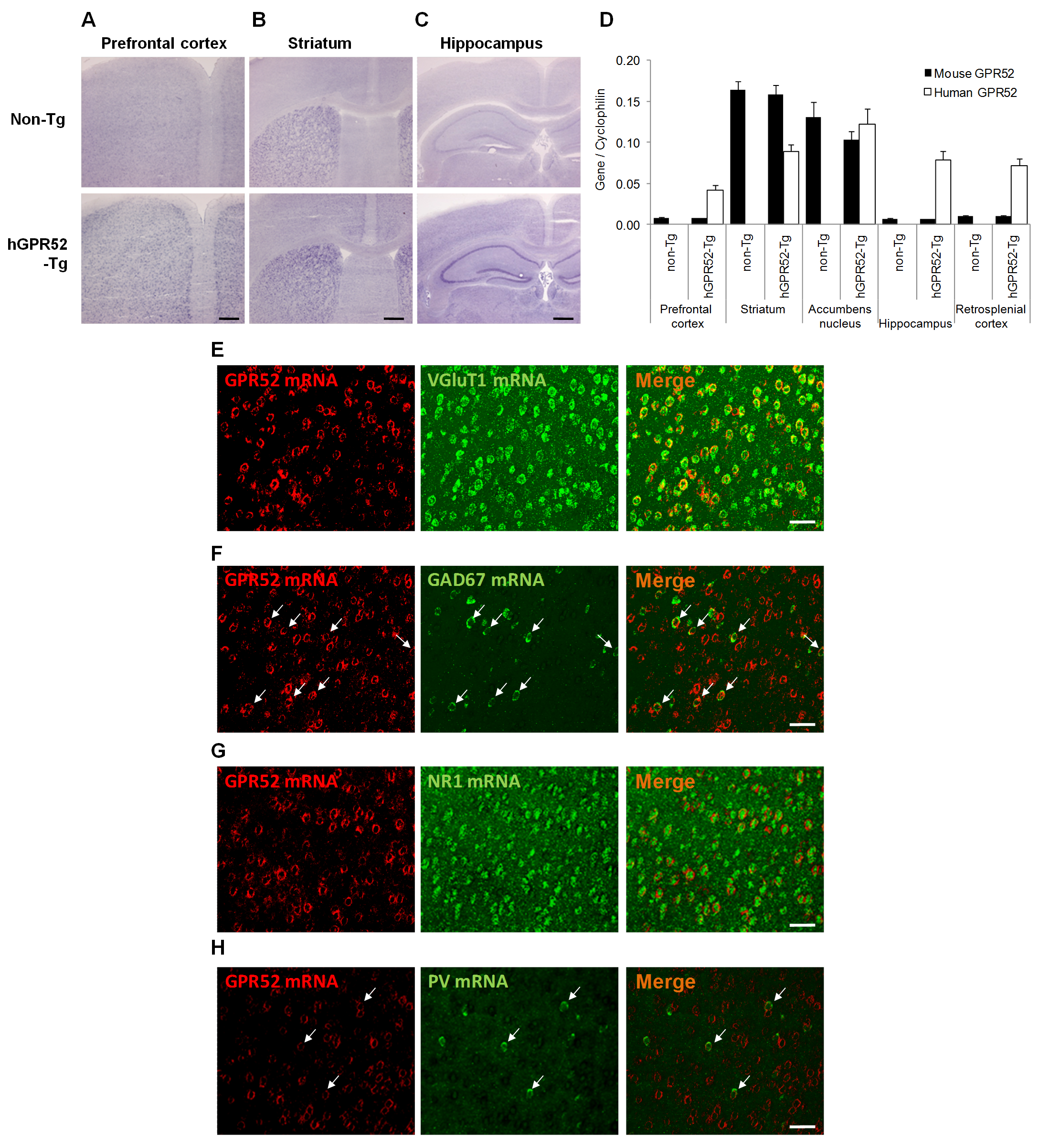

Supplement: Figure S7 — Characterization of GPR52-expressing neurons in hGPR52 Tg mice. A–C, GPR52 mRNAs were detected by ISH using hGPR52 cRNA probe in prefrontal cortex (A), striatum (B), and hippocampus (C) in hGPR52 Tg mice. Bar: 0.25 mm (A), and 0.5 mm (B and C). Because the hGPR52 cRNA probe was crossreacted to endogenous GPR52 mRNA in the non-Tg mice, the signals were significantly more increased in hGPR52-Tg mice than in non-Tg mice. D, Gene expression levels of hGPR52 and endogenousGPR52 (mGPR52) in brain were examined by qPCR. mGPR52 and hGPR52 mRNA levels were normalized by cyclophilin. Mean ± SEM (n = 5). The hGPR52 mRNA was highly expressed in the hGPR52-Tg mice while the endogenous GPR52 (mGPR52) mRNA showed similar expression levels in hGPR52-Tg and non-Tg mice. E–H, Double-ISH investigation of GPR52 with VGluT1 (E), GAD67 (F), NR1 (G), and PV (H) in prefrontal cortex of hGPR52 Tg mice. Red shows GPR52 and green in each picture shows VGluT1, GAD67, NR1, and PV. Arrows show double positive neurons. In (F) and (G), almost all of the GPR52-expressing neurons expressed the VGluT1 and the NR1 mRNAs. Bar: 100 µm. (TIF) [file pone.0090134.s007.tif]

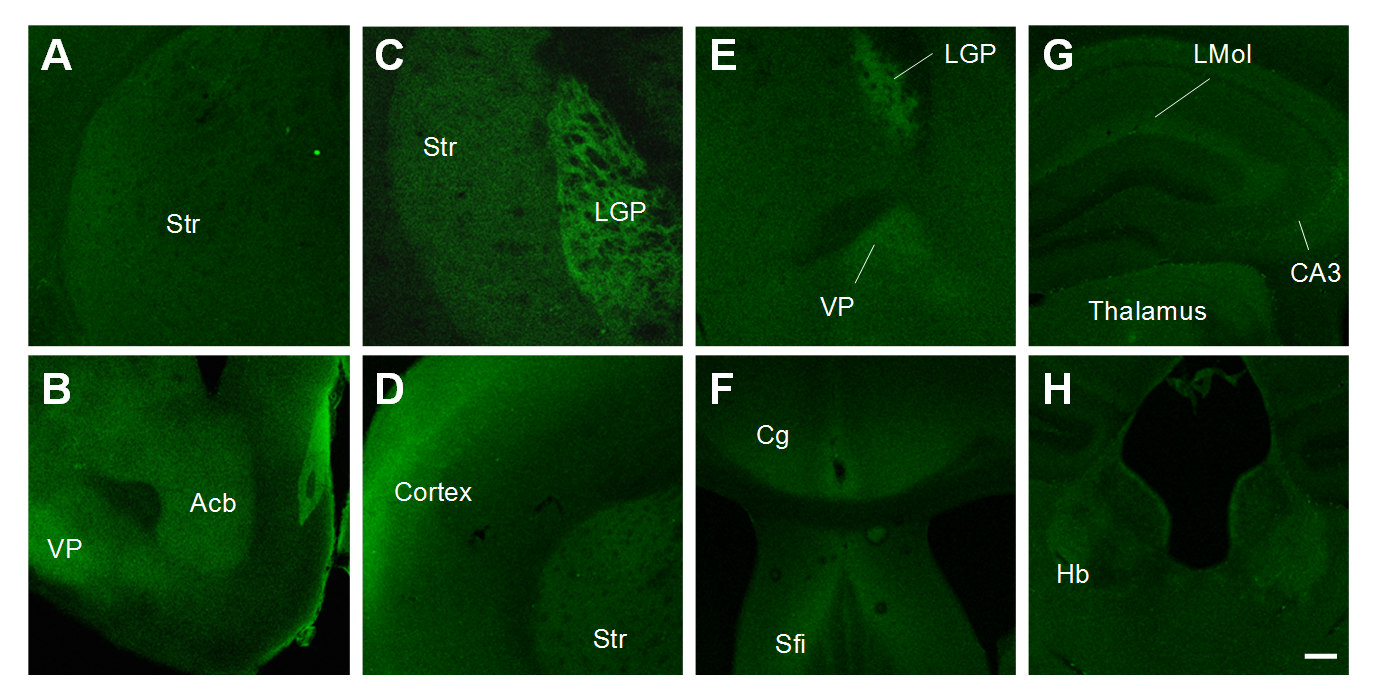

Supplement: Figure S8 — GFP fluorescence in brain of hGPR52-GFP Tg mice. A–H, GFP signals in coronal brain sections of hGPR52-GFP Tg mice were detected by confocal microscopy. All of the GFP signals were observed in fibers, but not in cell bodies. Abbreviations were shown in Table 2. Bar: 200 µm. (TIF) [file pone.0090134.s008.tif]

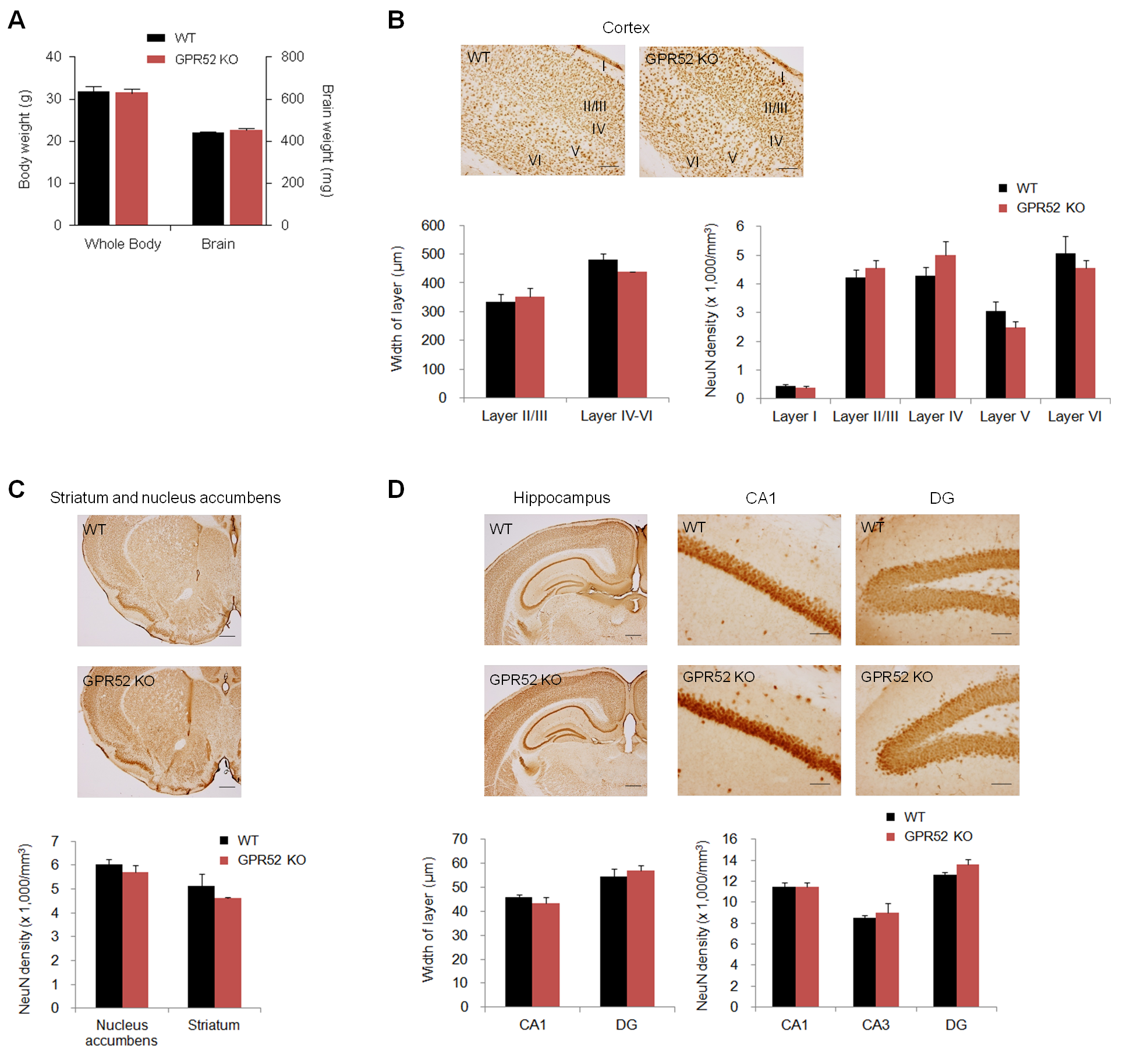

Supplement: Figure S9 — Brain morphology of GPR52 KO mice. A, Whole body and brain weights in GPR52 KO mice and WT littermates (5-month-old males). Mean±SEM (n = 8). B–D, Brain slices were stained using the NeuN antibody. B, Pictures show representative cortical sections. Scale bar, 100 µm. Bar graphs indicates widths of the layers (upper) and NeuN density (lower) in cortex. Mean±SEM (n = 3). C, Pictures show representative brain slices around nucleus accumbens and striatum. Scale bar, 400 µm. Bar graphs indicate NeuN density in those regions. Mean±SEM (n = 3). D, Pictures show representative brain slices of CA1 and dentate gyrus (DG) in hippocampus. Scale bar, 400 µm (left) and 50 µm (middle and right). Bar graphs indicate widths of pyramidal cell layer of CA1 and DG as well as NeuN density. Mean±SEM (n = 3). (TIF) [file pone.0090134.s009.tif]
